# Integrating thousands of PTEN variant activity and abundance measurements reveals variant subgroups and new dominant negatives in cancers

**DOI:** 10.1186/s13073-021-00984-x

**Published:** 2021-10-14

**Authors:** Kenneth A. Matreyek, Jason J. Stephany, Ethan Ahler, Douglas M. Fowler

**Affiliations:** 1 Department of Genome Sciences, University of Washington, Seattle, WA USA; 2 Department of Pathology, Case Western Reserve University School of Medicine, Cleveland, OH 44106 USA; 3 Present Address: Revolution Medicines, Redwood City, CA 94063 USA; 4 Department of Bioengineering, University of Washington, Seattle, WA USA

**Keywords:** PTEN, Protein variants, Multiplex assays of variant effect, Cancer genomics, Dominant-negative, VAMP-seq

## Abstract

**Background:**

PTEN is a multi-functional tumor suppressor protein regulating cell growth, immune signaling, neuronal function, and genome stability. Experimental characterization can help guide the clinical interpretation of the thousands of germline or somatic PTEN variants observed in patients. Two large-scale mutational datasets, one for PTEN variant intracellular abundance encompassing 4112 missense variants and one for lipid phosphatase activity encompassing 7244 variants, were recently published. The combined information from these datasets can reveal variant-specific phenotypes that may underlie various clinical presentations, but this has not been comprehensively examined, particularly for somatic PTEN variants observed in cancers.

**Methods:**

Here, we add to these efforts by measuring the intracellular abundance of 764 new PTEN variants and refining abundance measurements for 3351 previously studied variants. We use this expanded and refined PTEN abundance dataset to explore the mutational patterns governing PTEN intracellular abundance, and then incorporate the phosphatase activity data to subdivide PTEN variants into four functionally distinct groups.

**Results:**

This analysis revealed a set of highly abundant but lipid phosphatase defective variants that could act in a dominant-negative fashion to suppress PTEN activity. Two of these variants were, indeed, capable of dysregulating Akt signaling in cells harboring a WT PTEN allele. Both variants were observed in multiple breast or uterine tumors, demonstrating the disease relevance of these high abundance, inactive variants.

**Conclusions:**

We show that multidimensional, large-scale variant functional data, when paired with public cancer genomics datasets and follow-up assays, can improve understanding of uncharacterized cancer-associated variants, and provide better insights into how they contribute to oncogenesis.

**Supplementary Information:**

The online version contains supplementary material available at 10.1186/s13073-021-00984-x.

## Background

Genome and exome sequencing has revealed that individuals collectively harbor millions of germline and somatic protein-coding variants [1, 2]. Interpretation of the functional consequence of each observed variant is a major bottleneck for personalized genomic medicine. Missense variants are particularly challenging to interpret, as only ~2% of all presently reported germline missense variants have clinical interpretations [3], and a conclusive understanding of the disease-causing potential of the vast majority of both germline and somatic missense variants is missing [4]. Computational approaches are useful, but lack the high accuracy needed to confidently interpret the impacts of a given protein variant in a clinical setting. Thus, experimental characterization of protein variants in model systems offers a powerful method to aid in interpreting the impacts of human variants.

Traditional experimental assays that measure variant effects one at a time lack the throughput needed to characterize the thousands of missense variants that are possible within each disease-relevant protein. Multiplexed assays of variant effects (MAVEs), such as deep mutational scanning (DMS), resolve this bottleneck by measuring the effects of thousands of variants on a particular function or cellular property simultaneously [5]. Unfortunately, many disease-related proteins have multiple functions, and, generally, no single large-scale variant effect dataset can capture all of these functions. Thus, multiple distinct large-scale variant effect datasets may be needed to accurately phenotype variants in such proteins.

For example, PTEN is a multi-functional, disease-relevant protein where individual assays insufficiently capture the effects of its coding variation [6]. The *PTEN* gene encodes a 403 amino acid tumor suppressor protein, whose lipid phosphatase activity catalyzes the conversion of the growth-promoting phospholipid PtdIns(3,4,5)P_3_ into the alternative form PtdIns(4,5)P_2_. PTEN also has both protein phosphatase-dependent and -independent roles in genome maintenance, DNA repair, and cellular morphology [6]. Accordingly, germline *PTEN*-coding variation is associated with a collection of developmental abnormalities including Cowden Syndrome (MIM: 158350), grouped under the umbrella term *PTEN* Hamartoma Tumor Syndrome (PHTS). *PTEN* germline variation is also associated with macrocephalic autism spectrum disorder (MIM: 605309), and its pleiotropic effects can cause other phenotypes such as immune dysfunction. Somatic variation in *PTEN* is common in diverse cancers [7]. Despite intense study, the mechanism by which changes to each of PTEN’s functions impact its various roles and contribute to each disease remains unclear [8].

A pair of recent MAVEs, each measuring a separate property of PTEN, enabled the classification of large numbers of PTEN variants according to each property. We used variant abundance by massively parallel sequencing (VAMP-seq) to measure the steady-state abundance of 4112 PTEN missense variants when overexpressed in cultured human-derived cell lines [9]. Simultaneously, a separate group measured the lipid phosphatase activity of 7244 PTEN missense variants when overexpressed in yeast [10]. Both studies described how perturbations of the PTEN properties they measured correlated with disease, and both functional datasets have had implications for variant classification, either by comparing the data with the results of computational variant effect predictors to find discrepancies [11], or using the data to develop a better PTEN-specific predictor of variant pathogenicity [12].

Here, we use VAMP-seq to measure 764 additional variant abundance scores that were not previously measured, and add additional data to 3351 variants that were. We use this more comprehensive PTEN abundance dataset to explore the mutational patterns governing PTEN intracellular abundance and then incorporate the phosphatase scores to subdivide PTEN variants into four functionally distinct groups. This analysis revealed a set of highly abundant but lipid phosphatase defective variants that could act in a dominant-negative fashion to suppress PTEN activity. We validated that two of these variants are, indeed, capable of dysregulating Akt signaling in cells harboring a WT PTEN allele. Both variants were observed in multiple breast or uterine tumors cataloged in cancer genomics databases, demonstrating the disease relevance of a subset of high abundance, inactive variants, identifiable by analyzing paired, large-scale activity and abundance data. Thus, we show that multidimensional, large-scale variant functional data, when combined with public cancer genomics datasets and follow-up assays, can improve understanding of uncharacterized cancer-associated variants and provide better insights into their characteristics contributing to oncogenesis.

## Methods

### Plasmids

The pCAG-NLS-HA-Bxb1 (Addgene #51271) plasmid was a gift from Dr. Pawel Pelczar (University of Zürich, Switzerland). The attB-PTEN-HA-IRES-mCherry plasmid containing the PTEN cDNA sequence (NCBI accession number NM_000314.8) was modified by inverse PCR and Gibson assembly [13] to create the various variants tested for phospho-Akt1 Western blotting (Additional file 1: Table S1).

### Secondary PTEN Library generation

We previously determined that the biggest contributor to the sparseness of the original abundance dataset was loss of protein variants during the library generation process [9]. Thus, we sought to create a secondary, complementary library capable of supplying variants that were missing in the original library. We identified codons from the original PTEN site saturation mutagenesis library with five or fewer of the 20 possible protein-coding codons and re-amplified the sparsest 192 positions. We used a more permissive set of amplification parameters and higher cycle numbers to optimize coverage in these weakly represented codons. In individual tubes for each codon, 135 pg of attB-EGFP-PTEN-IRES-mCherry-562bgl plasmid was used in a 10 μL Kapa HiFi reaction with a final concentration of 0.5 μM forward and reverse primers and a final concentration of 5% dimethyl sulfoxide (DMSO). These reactions were denatured for 3 min at 95°C and cycled 25 times at 98°C for 20s, 52°C for 15s, and 72°C for 3 min with a final 3 min extension at 72°C. We ran 2 μL of each 10 μL reaction on a 1% agarose gel with SYBR Safe at 130V for 30 min. ImageJ was used to quantify the amounts of correctly sized amplicons for each codon. The ImageJ quantities were used to make an equal mix of all the codon amplicons.

The mix was cleaned and concentrated using a DNA Clean & Concentrator-5 kit (Zymo Research). The cleaned product was then phosphorylated using T4 PNK (NEB) and subsequently ligated using T4 ligase (NEB). The wild type plasmid template was digested using DpnI (NEB). The final ligated product was cleaned and concentrated again and transformed using 10-beta electromax cells (NEB). Small samples of a final 50 mL culture (before doubling could occur) were taken and plated in order to assess an approximate size of transformation. We chose to move forward with a library containing ~17,500 transformants. In order to get rid of small background plasmid, we moved the library into the final attB-EGFP-PTEN-IRES-mCherry-562bgl-KanR vector encoding kanamycin resistance using restriction enzymes XbaI and EcoRI-HF (NEB). Barcodes were added as previously performed [9], using a primer set ordered from IDT and filled in using Klenow (-exo) (NEB). The barcoded primer was inserted using SacII and EcoRI-HF (NEB). The final library was prepared for PacBio sequencing by digesting the ORF and associated barcode using SacII and XbaI (NEB) and processed using the PacBio Template Prep Kit 1.0 (Pacific Biosciences).

### Illumina sequencing of the library plasmid

Barcode sequencing of the plasmid library was also generated as previously performed [9]. Briefly, 50 ng of final midi-prepped (Sigma-Aldrich) plasmid was amplified and adapters were added in technical duplicate in 50 μL Kapa HiFi reactions (Roche). These reactions were denatured for 3 min at 95°C and cycled 5 times at 98°C for 20s, 60°C for 15s, and 72°C for 15s with a final 3 min extension at 72°C. The adapter reactions were cleaned using AMPure XP beads (Beckman-Coulter). Individual indices and Illumina cluster generating sequences were added in 50 μL Kapa Robust reactions (Roche). These reactions were denatured for 3 min at 95°C and cycled 25 times at 95°C for 15s, 60°C for 15s, and 72°C for 15s with a final 3 min extension at 72°C. The technical duplicates were mixed volumetrically, ran on a 1% agarose gel with SYBR Safe, and gel extracted using a freeze and squeeze column (Bio-Rad). The product was quantified using the Kapa Illumina quant kit (Roche) and sequenced on a NextSeq 500 using a NextSeq 500/550 High Output v2 75 cycle kit (Illumina). Sequencing reads were de-multiplexed and converted to FASTQ format with bcl2fastq. Barcode paired sequencing reads were joined using the fastq- join tool within the ea-utils package [14] using the default parameters. FASTQ files from these technical replicate amplification, and sequencing runs were concatenated for analysis with Enrich2 [15]. Illumina sequencing revealed there were ~ 33,274 unique barcodes within this library.

### Variant expression and sorting

All cell culture reagents were purchased from ThermoFisher Scientific unless otherwise noted. The HEK 293T LLP-iCasp9-Blast clone 12 cell line [16] was used for all the cell culture experiments. These cells were cultured in Dulbecco’s modified Eagle’s medium supplemented with 10% fetal bovine serum, 100 U/mL penicillin, and 0.1 mg/mL streptomycin. For induction of expression from the Tet-inducible promoter, the media were supplemented with 2 μg/mL Doxycycline (Sigma). Cells were detached for routine passaging with 0.25% trypsin-ethylenediaminetetraacetic acid.

For library experiments, six-million total HEK 293T LLP-iCasp9-Blast clone 12 cells were transfected over four 6-well plates. Each 6-well was seeded with 250,000 293T cells and mixed with 1500 ng pCAG-NLS-Bxb1, 1500 ng attB-EGFP-PTEN-IRES-mCherry-562bgl Fill-in library plasmid, and 6 μL Fugene 6 (Promega), in doxycycline-free media. Two days following transfection, the media were switched to Dox-containing media. The next day, AP1903/Rimiducid (MedChemExpress) was added to a final concentration of 10nM. The media were exchanged the next day to remove dying cells. Surviving cells were pooled into T175 plates and passed for roughly 1 week, when a fraction of the selected cells were frozen for storage. The remaining cells were propagated and processed for fluorescence activated cell sorting.

The library sorting experiments were performed as previously described [9]. Briefly, recombined cells were lifted from the plates with Versene solution (0.48 mM EDTA in PBS) to minimize clumping experienced with Trypsin digestion. The cells were pelleted at 300x*g* for 3 min and resuspended in sorting buffer (1X PBS + 1% heat-inactivated FBS, 1 mM EDTA and 25 mM HEPES pH 7.0) and filtered through a 35-μm nylon mesh. The cells were sorted on a BD FACSAria III machine using a 85-μm nozzle and equipped with FACSDiva software. mTagBFP2 fluorescence was excited with a 405-nm laser and detected after passing through a 450/50 nm band pass filter. EGFP fluorescence was excited with a 488-nm laser and detected after passing through a 505-nm long pass and 530/30-nm band pass filters. mCherry fluorescence was excited with a 561-nm laser and detected after passing through a 600-nm long pass and 610/20 band pass filters. All events had to pass through a FSC-A and SSC-A live cell gate, followed by a FSC-A and FSC-H gate for identifying singlets. Recombinant cells were isolated by gating for mTagBFP2 negative, mCherry positive cells. Cells passing these gates were finally passed into a FITC:PE–Texas Red ratiometric parameter in the BD FACSDIVA software, where gates were drawn to separate the cells into equally populated quartiles based on their ratio. Three replicate integrations were conducted and sorted for recombinants (Additional file 1: Table S2).

### Illumina sequencing of sorted cells

Amplification and sequencing of the barcodes from the genomically integrated library were also performed as previously described [9]. Genomic DNA including the recombined plasmid was isolated from cells using Qiagen DNeasy columns with the addition of RNAse-A (ThermoFisher Scientific) in the first incubation step. The genomic DNA was first amplified using one primer 5′ of the Bxb1 recombination site and the adapter primer 3′ of the barcode in order to guarantee that unrecombined plasmids would not be amplified. These were 50 μL technical duplicate reactions in Kapa Hifi (Roche) containing ideally, but no more than 2.5 μg of genomic DNA. These reactions were denatured for 3 min at 95°C and cycled 5 times at 98°C for 20s, 65°C for 15s, 72°C for 2 min with a final 3-min extension at 72°C. The adapter reactions were cleaned using AMPure XP beads (Beckman-Coulter). Individual indices and Illumina cluster generating sequences were added in 50 μL Kapa Robust reactions (Roche) using SYBR Green I on a BioRad MiniOpticon. These reactions were denatured for 3 min at 95°C and cycled 20 times at 95°C for 15s, 60°C for 15s, and 72°C for 15s with a final 3-min extension at 72°C. The indexed amplicons were mixed based on relative fluorescence units, run on a 1% agarose gel with SYBR Safe, and gel extracted using a freeze and squeeze column (Bio-Rad). The product was quantified using the Kapa Illumina quant kit (Roche).

### Replicate filter

We tested a replicate filter (code found at https://github.com/MatreyekLab/PTEN_composite) [17] wherein a variant had to be observed in a minimal number of replicates to pass the filter. To determine the ideal value that should be used for this replicate filter, we performed a test where we sequentially tested all possible values of the replicate filter (0 through 15). Only variants observed at or above the replicate filter value were considered to have passed the filter, and variants observed in fewer replicates than the filter value were removed. For each replicate filter threshold value, we asked whether the upper bound of the 95% confidence interval of the nonsense variant score overlapped with the lowest 5% of synonymous variant scores. If not, then it was deemed correctly scored. As a control, we resampled the synonymous and nonsense variant scores, but randomized the score associations with the number of replicates in which the score was a product of. A bootstrapping procedure was used to repeat this process 100 times per replicate filter value. The code used to perform this process is provided in the Github repository. A data table of all PTEN variant abundance scores is included as Additional file 2: Table S3.

### Combining abundance and activity scores

For lipid phosphatase activity, we restricted our analysis to variants classified as WT-like phosphatase activity, corresponding to the lower 95th percentile for synonymous variants (activity scores above 10^-1.11^), and loss of activity, corresponding to the upper 95th percentile for nonsense variants (activity scores below 10^-2.13^). Both values were used as cutoffs in the original manuscript [10]. To ease comparison with the abundance data, the phosphatase score was rescaled so that the mean nonsense variant score was set to zero, and WT was set to 1.

### Accession of public data

PTEN missense and nonsense variants were accessed from the ClinVar website on April 15, 2021, and manually filtered for single-codon changes. PTEN variants associated with autism spectrum disorder (ASD) were accessed from the SFARI Gene platform on April 15, 2021, and filtered for single codon changes [18]. PTEN missense and nonsense variants found in the population were accessed from GnomAD v2.1.1 (non-TOPMed) [1] on April 20, 2021, as well as from the TOPMed Freeze 8 with the Bravo database (https://bravo.sph.umich.edu/freeze8/hg38/) [19] on April 20, 2021. For the somatic variant analyses, PTEN missense and nonsense variants were taken from the “curated set of non-redundant studies” selection on cBioPortal [20, 21], which are drawn from 184 studies, corresponding to 48,035 samples. These were manually curated from both TCGA [2] and non-TCGA studies and do not have overlapping samples. We also drew samples from the separate AACR GENIE database [22] (also accessed through cBioPortal), corresponding to an additional 4 studies with 106,908 patients and 115,754 samples. The Cleveland Clinic Cohort data used in this work were calculated from the supplementary tables published by Mighell et al. [12]. As noted in the original work, the CC cohort consists of 256 prospectively accrued individuals with germline PTEN nonsynonymous variants, with 145 individuals encoding missense variants, and the remaining 111 encoding nonsense variants.

### Western blotting

HEK 293T LLP-iCasp9-Blast clone 12 [16] cells were transfected with the 1.5 μg pCAG-NLS-HA-Bxb1 and 1.5 μg of the attB-PTEN-HA-IRES-mCherry plasmids encoding WT PTEN or a PTEN variant using 6 μL of Fugene 6, in the absence of doxycycline. Four days after transfection, the cells were switched to doxycycline-containing medium, and 10 nM of AP1903 was added to kill off un-recombined cells. Each confluent 6-well was collected with 0.25% Trypsin-EDTA, washed in PBS, and incubated with 50 μL of lysis buffer (20 mM Tris pH 8.0, 150 mM NaCl, 1% Triton X-100, and Protease Inhibitor Cocktail (Sigma-Aldrich)) for 10 min at 4°C. The lysed cells were spun at 21,100x*g* for 10 min at 4°C, and the supernatant was collected into a separate tube. 12 μL of cleared lysate was mixed with 4 μL of 4x sample loading dye and separated on a NuPage 4–12% Bis-Tris gel (Invitrogen) in MOPS buffer, using Spectra Multicolor Broad Range Protein Ladder (ThermoFisher Scientific). The separated proteins were transferred onto a polyvinylidene difluoride membrane using a GENIE® Electrophoretic Transfer cassette (Idea Scientific). The membranes were blocked overnight in 5% milk powder in tris-buffered saline (20 mM Tris, 150 mM NaCl) with 0.1% Tween-20.

Each membrane was Western blotted with a 1:2000 dilution of anti-phospho-AKT (p.Thr308; 13038; Cell Signaling Technology) followed by detection with a 1:4000 dilution of anti-rabbit-HRP (NA934V; GE Healthcare), a 1:2000 dilution of anti-pan-AKT (2920; Cell Signaling Technology) followed by detection with a 1:5000 dilution of anti-mouse-HRP (NA931V; GE Healthcare), 1:5000 dilution of anti-HA-HRP (3F10; Roche), or a 1:2000 dilution of anti-beta-actin-HRP (ab8224; Abcam), using the SuperSignal West Dura extended duration substrate (ThermoFisher Scientific).

### Statistical analysis

Pearson’s *r* and Spearman’s *ρ* were calculated using the base functions in R. The linear models used in the work were calculated using the lm() base function in R. The permutation test used in the bootstrapped replicate filter analysis is described above and is encoded and fully replicable with the R Markdown file found at the aforementioned Github repository https://github.com/MatreyekLab/PTEN_composite [17].

The null distributions of PTEN variant subsets in the cancer genomics data were determined without using cancer-specific mutational signatures, as we previously observed little effect on the distribution of abundance scores when cancer-specific transition and transversion rates were used to estimate the null distribution [9]. To create the null distribution, a Python script was used to computationally substitute every nucleotide within the PTEN cDNA to every other nucleotide, resulting in a list of all possible single-nucleotide variants, and each of these codons was computationally translated. Synonymous variants were removed from the list. The list was then collapsed to unique missense and nonsense variants, which were also given frequencies of representation based on the degeneracy of the codon table. This table of missense and nonsense variant frequencies was subsequently split into subclasses based on each variant’s abundance and activity phenotypes, and the frequencies of the variants were added to provide the null estimate of expected class frequency in the absence of selection.

## Results

### Mutational tolerance patterns for PTEN abundance

We previously developed VAMP-seq, a generalizable method to simultaneously measure the effects of thousands of missense variants of a protein on intracellular abundance [9]. In VAMP-seq, each cell expresses a different protein variant directly fused to a fluorescent protein such as the enhanced green fluorescent protein (EGFP), so each cell’s level of fluorescence is directly proportional to the steady-state abundance of that protein variant. Single-copy, site-directed genomic integration into a HEK 293T landing pad cell line permits the expression of a library of thousands of protein variants in a pooled format [23]. The pooled cells are then separated into four bins of graded fluorescence using fluorescence-activated cell sorting (FACS). High throughput DNA sequencing is used to quantify the distribution of each variant across the four bins, and this distribution is analyzed to yield an abundance score for each variant. VAMP-seq has thus far been applied to five proteins: PTEN [9], TPMT [9], NUDT15 [24], VKOR [25], and CYP2C9 [26].

**Fig. 1:**
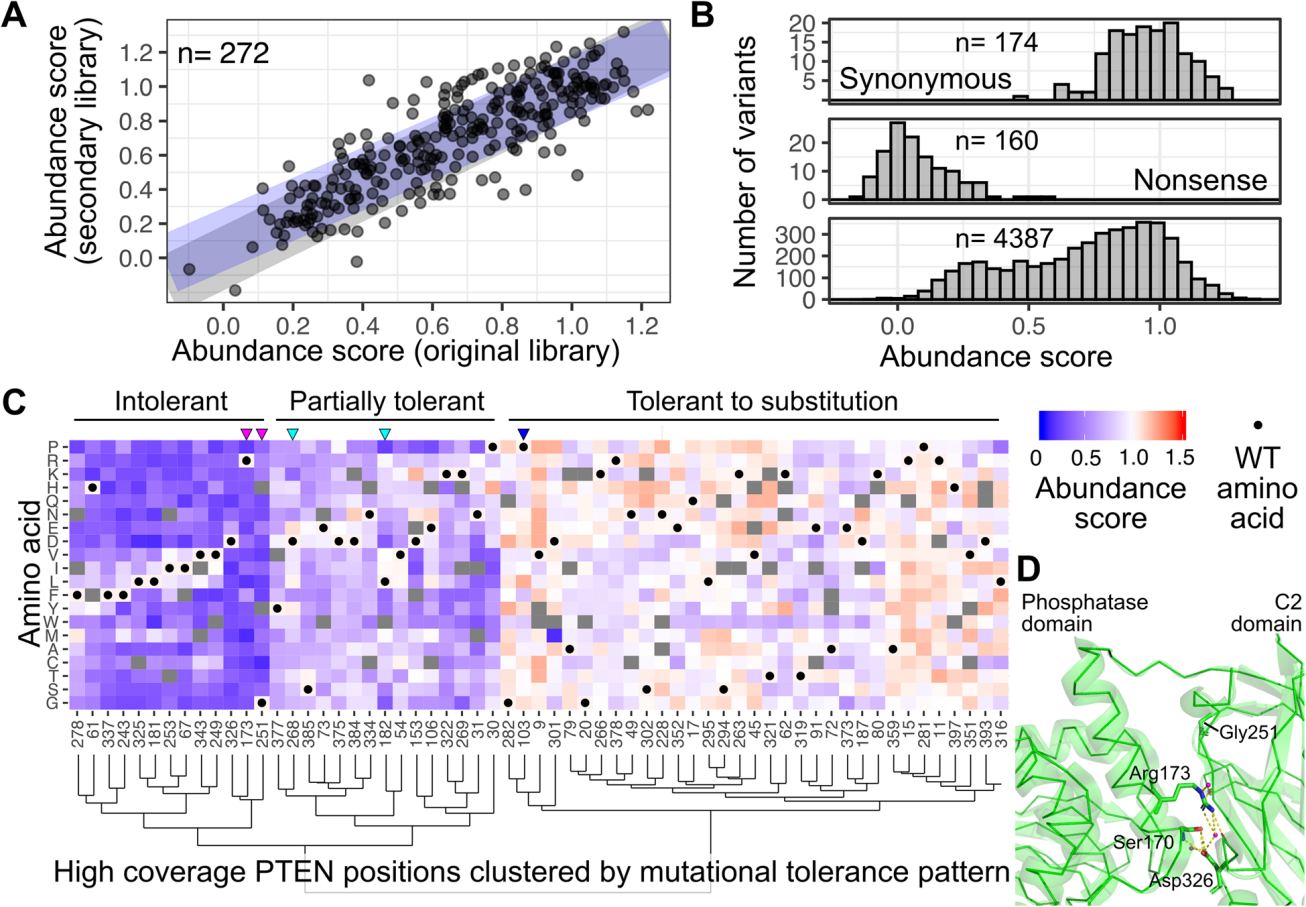
Steady-state cellular abundance data for 4721 PTEN variants. **A** Variants observed in 5 or more replicate in both the original and new PTEN abundance libraries were scored independently and compared. Gray line: ideal correlation with a slope of 1 and intercept of 0. Purple line: Observed correlation with a slope of 0.9 and intercept of 0.1. **B** Abundance scores for all subsets of variants observed in 4 or more replicates of the combined dataset. **C** PTEN high-coverage positions with 17 or more scored missense variants were assessed for their mutational tolerance patterns and shown in a clustered heatmap colored by abundance score. Variants that were not scored are colored in gray. WT residues are identified by black dots. Positions were ordered with hierarchical clustering based on their mutational tolerance patterns, with the corresponding dendrogram shown on the bottom. **D** Ball and stick representations of the Arg173 and Asp326 side chains on the PTEN crystal structure (1d5r), with the hydrogen bonding network at the PTEN inter-domain boundary causing Arg173 and Asp326 to be completely intolerant to substitution. Nitrogen atoms are colored in blue. Oxygen atoms are colored in red. Water molecules are colored in magenta. Predicted hydrogen bonds are denoted as yellow dashed lines. Gly251 is in a turn facing the interdomain interface and is shown at the top of this image

To supplement the 4407 PTEN variants whose abundance we measured initially [9], we generated a new PTEN library focused on positions with low coverage in the initial experiment (Additional file 1: Fig S1A, B). We re-amplified 198 low coverage positions using degenerate NNK primers, fused the resulting PTEN variant library to EGFP, and tagged each plasmid with a unique nucleotide barcode. We introduced the library into an improved HEK 293T landing pad cell line [14], which allowed for rapid enrichment for modified cells expressing the library by triggering apoptosis of the unmodified cells in the culture using the built-in iCasp9 cassette. We conducted seven replicate VAMP-seq experiments using this library, yielding additional abundance data for 4186 unique variants within this secondary library. 272 variants were observed in 5 or more replicates in both the initial and new experiments, and abundance scores for these overlapping variants were well correlated (Pearson’s *r*^2^ 0.70, Fig. 1A). A linear model fit to the scores of the overlapping variants had a slope of 0.89 and an intercept of 0.11, suggesting that the abundance scores from the two libraries could be combined without additional normalization steps.

Thus, we aggregated abundance scores from the initial and new libraries (Additional file 1: Fig S1C). We created a composite abundance score by taking the mean of all replicate scores for variants observed in 4 or more replicates, a threshold we set using a permutation test of the separation of synonymous and nonsense variants (Additional file 1: Fig S2) [17]. As expected, the number of variants encoded by two or more unique codons increased with the composite dataset (Additional file 1: Fig S3A). The final filtered dataset of 4721 variants included 174 synonymous variants, 160 nonsense variants, and 4387 missense variants including 764 variants not scored in the initial experiment (Fig. 1B). 3904 variants scored in the initial experiment received modestly refined scores, reducing the coefficient of variation for many variants (Additional file 1: Fig S3B). This resulted in a net 12.5% increase in the number of variants with a coefficient of variation less than 0.5 (Additional file 1: Fig S3C). We compared our revised scores to a set of 24 PTEN variants we had individually assessed for steady-state abundance in the original manuscript, and while the correlation coefficient did not change with the refined score, this is likely because the correlation with the original data was already high (Spearman *ρ*^2^ = 0.93; Additional file 1: Fig S3D). Altogether, the number of variants confidently assigned low abundance classifications increased from 1260 to 1423, and the number of variants confidently assigned WT-like classifications increased from 1577 to 1738 variants (Additional file 1: Fig S3E). Thus, as cell and library engineering approaches improve, VAMP-seq datasets can be bolstered with additional replicates containing new variants, and existing datasets can be reanalyzed to create more complete and accurate datasets.

An advantage of the composite abundance dataset was an increased number of positions with high variant coverage, revealing patterns of amino acid substitutions tolerated at each position. The composite dataset included 61 positions where approximately 90% of missense variants were scored (increased from an original 50 positions) and 22 positions that had full coverage (increased from an original 9 positions). We performed hierarchical clustering of the high coverage position abundance scores, which yielded groups of intolerant, partially tolerant, and tolerant positions (Fig. 1C). Roughly half (*n* = 33) of positions were nearly uniformly tolerant to substitutions. In contrast, 15 positions were partially tolerant to substitution, while the remaining 13 positions were intolerant. Residues Arg173, Gly251, and Asp326 were almost entirely intolerant to substitution (Fig. 1C). All three of these residues are located in the interface between the PTEN phosphatase and C2 domains, suggesting that specific characteristics of the WT amino acids at these positions are critical for keeping the interface intact. Consistent with this hypothesis, Arg173 and Asp326 make extensive polar contacts in the PTEN structure (Fig. 1D). With the exception of His61, the remaining intolerant positions encoded phenylalanine, isoleucine, leucine, or valine residues and were only partially tolerant of other bulky hydrophobic side-chains (Fig. 1C).

Missing data is often computationally imputed using machine learning to yield a complete dataset needed for certain downstream analyses [27, 28]. A recent study imputed missing PTEN abundance data prior to training a logistic regression classifier of PTEN variant effect and molecular phenotype [12]. 693 of the imputed abundance values were scored in our refined dataset, allowing an independent confirmation of the accuracy of the imputation. We examined the correlation between the imputed abundance data and newly acquired data from our second library and found moderate correlation and scaling between the imputed and experimentally determined values (slope: 0.53; Pearson’s *r*^2^: 0.54; Additional file 1: Fig S4). This was consistent with the correlation observed by the authors during 10-fold cross-validation of their initial imputation algorithm with the initial abundance dataset (Pearson’s *r*^2^: 0.56). The lower than expected slope is largely explainable by a difference in scaling of the imputed data, which ranged from 0.25 to 1, whereas the measured values ranged from 0 to 1.

We next examined positions which were poorly imputed to better understand the situations in which the imputation algorithm struggled to accurately predict abundance scores. Variants of Arg173 and Gly251, two of the three positions highlighted earlier as being almost entirely intolerant to substitution, were incorrectly imputed with intermediate abundance scores, likely because our analyses showed that these WT residues were unusually critical for maintaining PTEN abundance (Fig. 1C, magenta triangles). Imputed values for proline residues were generally low, even for residues such as Pro103 and Pro248, which were highly amenable to substitution and yielded numerous variants with WT-like scores (Fig. 1C, blue triangle). The algorithm also struggled with Tyr180, Leu182, and Asp268, which were residues that were partially tolerant to substitution and exhibited a wide range of abundance scores depending on the variant, whereas the imputed values roughly approximated the positional mean (Fig. 1C, cyan triangles). By providing additional experimental data and reducing reliance on imputation, the additional abundance data we furnish here will improve accuracy in the downstream uses of the abundance data.

### Classification of PTEN variants by abundance and activity

**Fig. 2:**
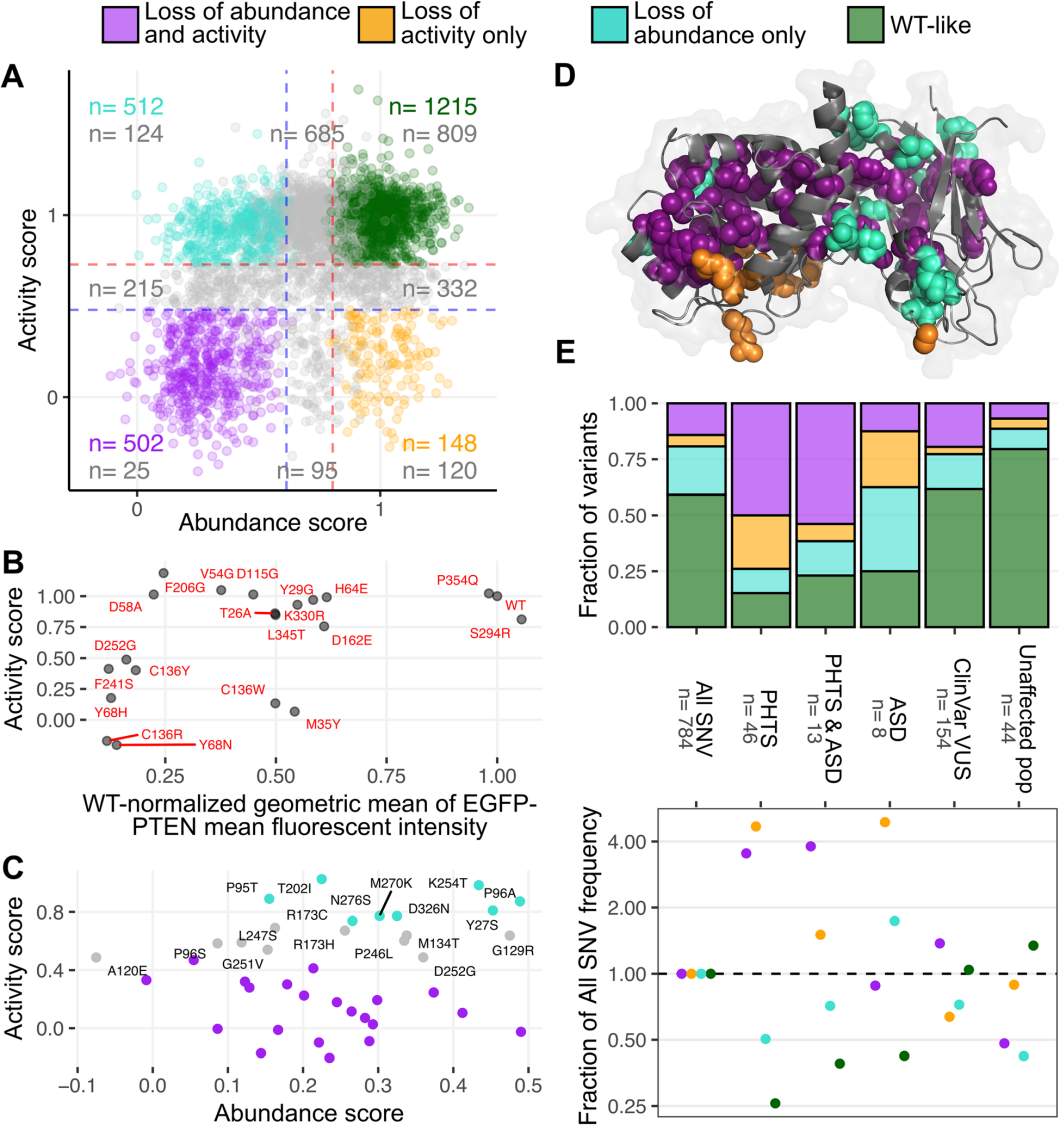
Four-way classification of PTEN variants. **A** Scatterplot of PTEN variants scored in both assays, with abundance scores shown on the *x*-axis, and phosphatase scores shown on the *y*-axis. WT-like variants are shown in green, loss of activity variants are shown in orange, loss of abundance variants are shown in cyan, and variants that have both losses in activity and abundance are shown in purple. The total counts of the classified or unclassified (gray) variants in each sector of the plot are shown. **B** Scatterplot of activity scores and individually assessed EGFP fluorescence for 20 variants. Variants at known catalysis affecting residues 45, 124, and 129, as well as PIP_2_ binding residues 1 through 13, were removed from the analysis. **C** Scatterplot of ClinVar pathogenic or CC cohort PHTS or autism spectrum disorder (ASD) variants with low abundance, plotted by abundance and activity scores. Variants that did not score as low for activity are labeled. **D** Positions with variants of extreme effects shown on the PTEN crystal structure (pdb: 1d5r). **E** (Top) Bar chart showing the distribution of the abundance and activity PTEN variant subsets across the various clinical groupings. (Bottom) The fraction of each variant class for each category was divided by the fraction of the corresponding variant class in the All SNV category to calculate the fold enrichment or depletion. The PHTS category includes ClinVar pathogenic or likely pathogenic PTEN variants for PHTS, and variants from patients identified with PHTS in the CC cohort. The ASD category includes variants listed in the SFARI Gene database, and variants from patients identified with ASD or developmental disorders in the CC cohort. Variants associated with both were put in their own category of PHTS & ASD. VUS are variants of uncertain significance in ClinVar. PTEN variants observed in unaffected populations captured by the GnomAD and TOPMed databases are also shown

Our composite abundance scores, along with the PTEN lipid phosphatase scores measured in yeast [10], provide two distinct measures of the properties of a total of 4178 PTEN missense variants. We integrated these two datasets to separate PTEN variants into four distinct subsets: WT-like, loss of abundance only, loss of activity only, and loss of both abundance and activity. To perform this four-way classification, we focused on variants that were confidently scored by both assays and thus could be categorized according to both properties (Fig. 2A; see Methods). The majority of variants were in agreement with both assays, as 51% of the classified variants were WT-like for both properties (Fig. 2A, green), while 21% exhibited loss of both activity and abundance (Fig. 2A, purple).

The remaining 28% of variants exhibited discrepancies between the two assays, pointing to subtler molecular phenotypes than near-complete losses of phosphatase activity through loss of intracellular abundance. The smallest subsets were the 6% of variants classified as loss of activity only variants, where phosphatase activity was abrogated without affecting protein steady-state abundance (Fig. 2A, orange). The remaining variants, accounting for 22% of the total classified variants, were loss of abundance only (Fig. 2A, turquoise). These are likely variants that have no inherent effect on phosphatase activity, yet may reduce the total amount of intracellular PTEN expressed in cells, such that the net result may be hypomorphic functioning in cells. This included Asp331Gly, which also exhibited reduced abundance when expressed in U87-MG glioblastoma cells [29], but had near-WT phosphatase activity when purified and equimolar amounts were tested in vitro [29, 30].

The preponderance of loss of abundance only variants prompted us to examine the relationship between the two assays more closely. We first analyzed the scaled activity scores of a panel of 20 variants exhibiting a range of abundances that had been individually assessed for their steady-state mean fluorescence intensity when fused with GFP [9]. The variants in this panel only consistently showed reduced activity when their measured abundances were profoundly reduced, by at least 5-fold (Fig. 2B). Thus, very low abundance variants can score as WT-like in the yeast rescue activity assay.

To better understand whether these low abundance variants without reduced yeast rescue activity scores were clinically important, we focused on the subset of low abundance PTEN variants classified as pathogenic in ClinVar, or associated with autism spectrum disorder (ASD) or PTEN Hamartoma Tumor Syndrome (PHTS) in a recently published cohort of well curated PTEN variant positive individuals seen at Cleveland Clinic (CC cohort) [12]. Of the 40 total variants in this set, 22 (55%) were classified as loss of both activity and abundance variants (Fig. 2C). The remaining 18 variants (45%) were confidently assessed as low in abundance, with 8 considered loss of abundance only due to their high activity scores. The other 10 exhibited intermediate activity. Notably, 12 of these clinically meaningful variants (Tyr27Ser, Gly129Arg, Met134Thr, Arg173Cys, Arg173His, Thr202Ile, Pro246Leu, Gly251Val, Asp252Gly, Lys254Thr, Asn276Ser, Asp326Asn) have reduced in vitro activity, reduced abundance, or altered function when assessed in human cell models by other labs, further supporting these variants’ perturbed function [30–38]. For the 6 remaining pathogenic variants, the low abundance score in our dataset is the only measurement of altered experimental consequence to date. Thus, it is tempting to speculate that at least some of these loss of abundance only variants are pathogenic by sole virtue of their lowered abundance despite scoring as WT-like or indeterminate in the yeast activity assay. However, it is also possible that noise in either assay, a lack of sensitivity to subtle but clinically meaningful loss of activity, or alterations in function not captured by the yeast activity assay, could be responsible for the pathogenicity of these variants.

We next asked how variants in each subset were distributed within the PTEN protein structure. At 42 positions, the majority of variants led to both loss of abundance and loss of activity, and these largely mapped to the buried regions of both PTEN domains (Fig. 2D, purple spheres). At 17 positions, the majority of variants led to a loss of abundance only, and these positions were located around the periphery of the PTEN structure, especially within the C2 domain (Fig. 2D, turquoise spheres). At 9 positions, variants led to a loss of activity only, and these largely mapped around the active site but included Ala333, a membrane-proximal residue on the C2 domain (Fig. 2D, orange spheres).

We then analyzed how each subset related to the variants found in publicly available databases for PHTS, ASD, and various tumors biopsied from cancer patients, or in the CC cohort [12]. We found 59 germline pathogenic or likely pathogenic variants associated with PHTS in ClinVar, or characterized as having classic PHTS symptoms in the CC cohort, that were also confidently scored in both abundance and activity datasets. Of these, 46 were PHTS only variants that were not observed associated with autism spectrum disorder or developmental disabilities in the SFARI database or the CC cohort. Compared to all possible single-nucleotide variants, or variants observed in unaffected population databases such as GnomAD or TOPMed, these PHTS only variants were enriched for loss of abundance and activity variants, as well as loss of activity only variants (Fig. 2E, purple and orange bars). In contrast, loss of abundance variants were slightly reduced, while WT-like variants were reduced (Fig. 2E, turquoise and green bars). There were 8 variants that were only associated with autism spectrum disorder. While the sample is small, there were enrichments in loss of abundance or loss of activity variants, but no enrichment in variants with concomitant losses to both abundance and activity. On the other hand, there were 13 germline variants associated with both classic PHTS symptoms and autism spectrum disorder. Seven of these variants were loss of both abundance and activity.

The enrichment of loss of activity and abundance variants in individuals with PHTS, particularly those also exhibiting autism spectrum disorders, was consistent overall with the phenotypes observed in a similar analysis by Mighell et al. [12]. There was a notable difference in the enrichments of loss of abundance only or loss of activity only variants. They observed more loss of abundance only variants associated with PHTS, whereas we observed more loss of abundance only variants associated with autism spectrum disorder. Their method differed from ours, as their analysis looked at frequencies of variants and variant classes observed within PTEN variant-positive individuals in their cohort, while we utilized a larger set of data where information on frequency was not always available. We thus examined how each abundance and activity class was populated by unique PTEN variants, and the aforementioned differences may be due to these methodological differences. Regardless, distinct clinical groups appear potentially enriched with different PTEN molecular phenotypes. In contrast, there were only slight enrichments and depletions observed in the 154 variants of uncertain significance (VUS) in ClinVar that were also confidently scored in both datasets. 52 of these variants exhibited either loss of abundance or loss of activity based on these criteria and may be prime targets for reclassification in the future.

Reclassifying these variants will require incorporation of PTEN-specific considerations for clinical interpretation by expert working groups [39]. Along these lines, the initial PTEN abundance and activity data were used to create a logistic regression model capable of separating clinically significant PTEN variants from other variants [12]. This model revealed that PTEN variants with intermediate activity in yeast or truncation-like missense variants were appreciably more likely to also cause PHTS, supporting the use of abundance and activity measurements as evidence of pathogenicity [12]. This study relied on imputed missing abundance scores to train their model. By providing additional experimental data and reducing reliance on imputation, the additional abundance data we provide here will empower refinement of PTEN variant reclassification.

**Fig. 3:**
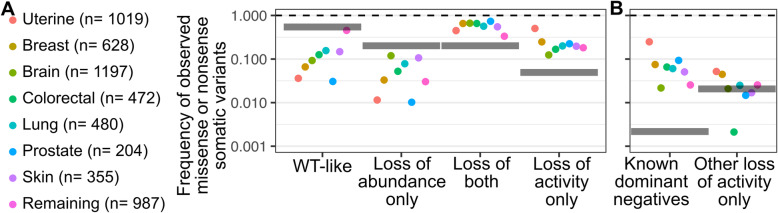
Distribution of somatic PTEN variants in cancer genomics datasets. **A** Each nucleotide within the PTEN cDNA was computationally substituted to every other nucleotide to create a list of all possible single-nucleotide variants, and each of these codons was computationally translated to create a list of PTEN missense variants possible through single-nucleotide variation, as well as their relative frequencies of being observed based on degeneracy of the codon table. Approximately 46% of these missense variants had clear abundance and activity classifications (this is the sum of all of the gray lines across the four classes shown in panel A). To aid comparisons for cancer-specific enrichment of PTEN variant classes, these PTEN variant frequencies calculated from mutation only were separated into different phosphatase-abundance groups, and their expected frequencies are shown as thick gray lines. PTEN variants observed in cancer genomics databases were also separated by phosphatase-abundance groups, and their frequencies shown as different colored points. **B** Loss of activity only variants were next separated into known dominant negatives, as well as other previously uncharacterized loss of activity only variants. The total number of somatic PTEN variants observed in cancer genomics databases with concomitant abundance and activity classifications, is shown as *n* values next to each cancer type

Next, we examined enrichment of variants in the different PTEN abundance and activity subsets in breast, uterine, lung, colorectal, prostate, skin, and brain cancers found in various cancer genomics datasets accessed with cBioPortal [20, 21]. Here, the goal was to use our data to better distinguish different types of potentially cancer-driving, PTEN loss-of-function variants, from the potentially innocuous PTEN variants that may have coincidentally accumulated during tumor development. We estimated a null model of mutation in the absence of selection by calculating the frequencies of each subset possible through single nucleotide variation (Fig. 3, grey bars). WT-like variants were uniformly depleted as compared to our null model, likely due to corresponding enrichments of variants from the other functionally damaging subsets outcompeting them. Loss of abundance only variants also appeared to be depleted, likely due to the fact that, by definition, these variants retain at least partial activity, which may be sufficient to counteract oncogenesis in most circumstances. In contrast, variants exhibiting both a loss of abundance and activity were uniformly enriched across cancer types. This finding is consistent with the enrichment of low abundance variants we previously observed [9] and suggests PTEN loss of function through loss of abundance along with loss of activity is a common contributor to oncogenesis across cancer types.

The remaining loss of activity only subset is particularly interesting as it includes dominant-negative PTEN variants [6]. Homodimerization is thought to keep PTEN in its active conformation and allow it to exert maximal PtdIns(3,4,5)P_3_ phosphatase activity [40]. Accordingly, cells encoding a WT PTEN allele exhibited greater Akt intracellular signaling when the dominant-negative variant Cys124Ser was co-expressed as compared to a null or destabilized variant [9, 40]. Consistent with this observation, transgenic mice where one allele was replaced with known dominant-negative alleles such as Cys124Ser and Gly129Glu exhibited increased tumor burden [40, 41]. Thus, we examined this subset for both known and potentially uncharacterized dominant-negative variants amongst the differing cancers.

Loss of activity only variants were differentially enriched across cancers, with breast, uterine, prostate, and lung cancers exhibiting the greatest enrichments (Fig. 3A). The known dominant-negative variants Cys124Ser, Gly129Glu, Arg130Gly, and Arg130Gln were observed at a much higher frequency than predicted by the null model, where they were collectively only possible through 5 of the 3618 single nucleotide-driven codon changes (Fig. 3B). These known dominant-negative variants were the major contributors to the enrichment of loss of activity only variants, contributing 58% of the enrichment observed in breast, 87% in uterine, and 100% in lung cancers. Thus, no additional loss of activity only lung cancer variants were scored, while uterine and breast cancers had enriched loss of activity only variants in addition to the known dominant negatives (Fig. 3B). We hypothesized that this additional loss of activity only variants observed in uterine and breast cancers might represent new dominant-negative variants.

### Identifying potential dominant-negative variants

**Fig. 4:**
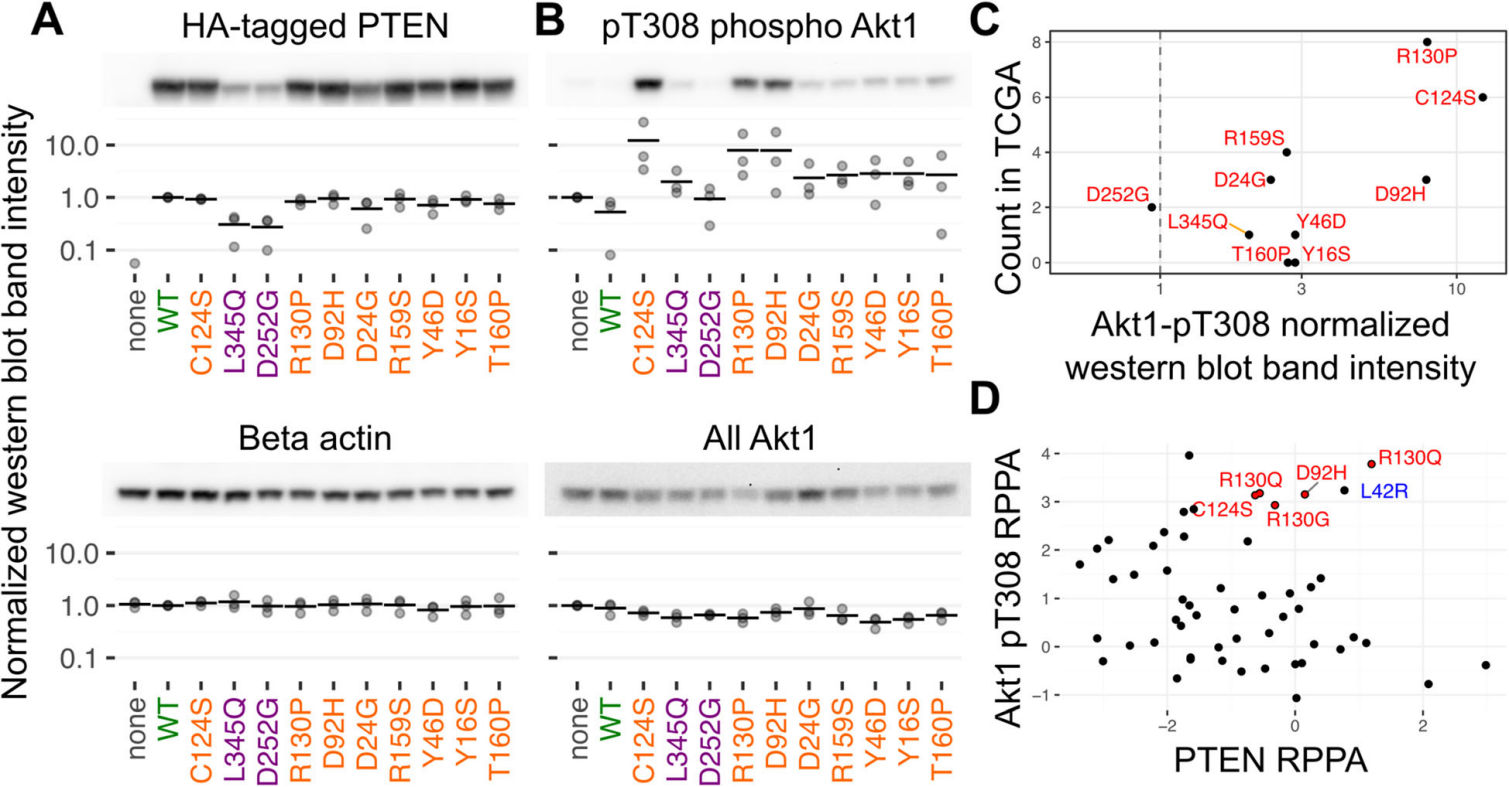
PTEN variants and phospho Akt1. WT or PTEN variants were overexpressed in HEK 293T cells, lysed, and Western blotted for the HA tag (**A**, top), Beta-actin (**A**, bottom), Thr308 phosphorylated Akt1 (**B**, top), or all Akt1 (**B**, bottom). Representative Western blots are shown. Normalized band intensities of each of three independent Western blotting experiments are shown as points, while the mean value is shown as a black bar. **C** Scatterplot comparing the mean normalized pThr308 value to the number of independent samples each variant was observed in the cancer genomics databases. **D** Comparison of PTEN protein abundance level and pThr308 Akt1 abundance level calculated by reverse-phase protein array in the cancer cell line encyclopedia. Leu42Arg, which was not tested in our assay, is highlighted in blue as it exhibits strong reverse-phase protein array data for both PTEN abundance and pThr308 in the CCLE data

To test this hypothesis and identify new PTEN dominant-negative variants, we quantified levels of Akt activation loop phosphorylation at Thr308 (pAkt) in cells expressing both a variant and a WT copy of PTEN [40] (Fig. 4, Additional file 1: Fig S5). We implemented this assay by expressing PTEN variants using our HEK 2393T landing pad cells, which already express WT PTEN [9]. Using this approach, we previously identified Pro38Ser as a dominant-negative variant, as it resulted in increased pAkt levels similar to the known dominant-negative Cys124Ser variant [9].

We chose a small panel of loss of activity only variants to screen using this assay including Asp24Gly, Asp92His, Arg130Pro, and Arg159Ser which were selected because they were observed multiple times in breast cancer, uterine cancer, or both. We also chose Tyr16Ser, Tyr46Asp, and Thr160Pro, which were observed one or zero times in tumors, allowing us to test whether observation in tumors correlated with dominant-negative activity. Finally, we included the known low abundance variants Leu345Gln and Asp252Gly, along with the known dominant-negative variant Cys124Ser, as controls.

As expected, all of the loss of activity only variants were expressed at near WT levels and were clearly distinguishable from Leu345Gln and Asp252Gly, the loss of abundance controls (Fig. 4A). Overexpression of WT PTEN reduced pAkt levels below that of unrecombined cells. In contrast, the loss of abundance control variants had pAkt levels similar to unrecombined cells, suggesting we could qualitatively distinguish variants presumably functional for PtdIns(3,4,5)P3 phosphatase activity over those that were inactive. Importantly, all of the loss of activity only variants exhibited pAkt levels equal to or greater than the unrecombined and loss of abundance controls, confirming these variants were, indeed, loss of activity. Along with the known dominant-negative variant Cys124Ser, the Arg130Pro and Asp92His variants exhibited markedly increased pAkt levels (Fig. 4B). This increase in pAkt signal was not due to elevated *AKT1* expression, suggesting a specific effect on Akt1 signaling. The remaining loss of activity only variants exhibited intermediate pAkt signals and were thus less conclusive.

Importantly, each variant’s ability to drive Akt1 Thr308 phosphorylation correlated with the variant’s incidence in cancers (Pearson’s *r*: 0.76) (Fig. 4C). Arg130Pro was found to be mutated four times in sequenced breast cancers, and three times in uterine cancers, and once in an esophageal cancer (Fig. 4C). Asp92His was mutated in three independent instances of breast cancer. Consistent with these results, the Asp92His variant is present in the CAMA-1 breast cancer cell line tested in the Cancer Cell Line Encyclopedia (CCLE) [42]. This cell line exhibits normal PTEN expression and elevated pAkt, similar to other cell lines expressing the known dominant-negative variants Arg130Gln, Cys124Ser, and Arg130Gly in CCLE [42] (Fig. 4D). In contrast, Asp24Gly and Arg159Ser, which were mutated two times in breast and uterine cancers, respectively, did not exhibit increased pAkt intensity in our assay. Thus, the PTEN large-scale variant functional datasets, when combined with cancer genomics data, can identify dominant-negative variants that may exhibit altered disease severity.

## Discussion

Here, we showed that integrating multiple large-scale PTEN variant functional datasets can reveal how variants impact protein function, and illuminate mechanisms by which disease-associated variants act. We improved our previous PTEN variant abundance dataset by assaying a new, independently generated library, adding abundance scores for 764 new variants. This was aided by newly engineered iCasp9 landing pad cells, which allowed us to rapidly select for our entire culture of recombined cells overnight. In contrast, the original method required FACS to sort for mCherry positive cells, which necessitated hours of preparation and sorting, costing many hundreds of dollars per replicate recombination. More importantly, by avoiding the FACS step through chemical selection, we could avoid library bottlenecking that occurs from extended culturing needed prior to sorting, the inability to capture every target cell during FACS, or the loss of cell viability that occurs from the sorting procedure. The combination of improved workflow, an independently generated library of previously missing variants, and slight improvement in accuracy of previously scored variants combined to yield a final dataset of 4721 variants confidently scored for abundance.

We integrated this improved abundance dataset with an existing large-scale PTEN phosphatase activity dataset to separate variants into four distinct subsets based on their abundance and activity. We compared the variants in these subsets to germline variants associated with PTEN hamartoma tumor syndrome or autism spectrum disorder. Disease-associated variants tended to have reduced activity, with the largest fraction of these also having reduced abundance. This finding is consistent with another recently published analysis comparing the PTEN variant activity and abundance, which reported that ASD and PHTS variants were enriched in loss of abundance and activity variants [12]. For PTEN somatic variants, we found that those with losses of activity and abundance were uniformly enriched across cancers, while loss of activity only variants were particularly enriched only in breast, uterine, and lung cancers. We analyzed four previously uncharacterized loss of activity only variants that were observed multiple times in breast cancer, uterine cancer, or both, and found that two of these variants exhibited increased pAkt signaling consistent with dominant-negative activity. These findings highlight the utility of having multiple, distinct large-scale variant functional datasets for important disease genes like *PTEN*, since integrating these data can reveal groups of variants that have different functional properties with different implications for disease.

As compared to null alleles, expression of certain cancer-associated PTEN variants in mouse models exacerbated developmental overgrowth and tumor burden [41]. These alleles also exhibited dominant-negative effects on the intracellular regulation of the PI3K/Akt pathway [40]. Thus, cancer-associated variants exhibiting dominant-negative in vitro activity are likely stronger drivers for cancer. Recent studies have begun to reveal differences in associations between some functionally distinct PHTS PTEN variants, particularly for their associations with autism, cancer, or both [33, 43]. However, additional research with larger cohorts is needed to fully characterize the genotype-phenotype associations of PTEN variants with different loss-of-activity levels, including dominant-negative variants.

Only Cys124Ser, Gly129Glu, Arg130Gly, and Arg130Gln were originally identified as exhibiting dominant negative in vitro activity [40]. We previously determined that Pro38Ser had WT-like abundance, but its recurrent appearance in melanomas suggested it could have been inactive and, thus, acting in a dominant-negative fashion [44]. Follow-up assays revealed that Pro38Ser overexpression in cells with a WT copy of PTEN drove abnormally high pAkt levels, supporting this notion [9]. Here, by comparing abundance and activity for thousands of PTEN variants, we directly identified numerous variants lacking activity while remaining abundant in the cells. These comprise the functional properties generally required for PTEN dominant-negative activity. Follow-up assays on four variants revealed that Arg130Pro and Asp92His also disrupt AKT regulation in the presence of a WT copy of PTEN and thus act in a dominant-negative fashion, at least in our cellular assay.

Arg130Pro joins Arg130Gly and Arg130Gln, two previously known dominant-negative variants affecting a key catalytic residue in the P-Loop/HCXXGXXR motif shared by protein tyrosine phosphatases and dual-specificity phosphatases [43, 45]. The observation that three different substitutions of the WT arginine residue can confer dominant-negative activity suggests that any stable PTEN variants lacking this side-chain are likely capable of interfering with WT PTEN activity. Variants with Arg130 substitutions to Ala, Phe, and Leu all scored as stable and inactive in our integrated analysis, and thus are highly likely to be dominant-negative variants as well. Of those three, only Arg130Leu is possible through single-nucleotide variation, and it is accordingly repeatedly observed in cancer genomics databases [22, 46].

Asp92 is a key residue in the PTEN WPD loop and is thought to coordinate and polarize the nucleophilic water molecule [47]. Like Gly129Glu, Asp92Ala was found to abrogate the removal of phosphate groups from phospholipids but not phosphopeptides [48]. However, Asp92His has not been biochemically characterized, so it is unclear whether it retains protein tyrosine phosphatase activity like Asp92Ala. Variants with Asp92 substitutions to Glu, Leu, Pro, Gln, Arg, and Ser all scored as stable and inactive in the integrated analysis, although only Asp92His and Asp92Glu are possible through single-nucleotide variation. Asp92Glu is observed in cancers even more often than Asp92His, suggesting that its negatively charged side-chain is not sufficiently able to mimic the role played by the aspartate and that it is also a dominant-negative variant. While not scored in our dataset, Asp92 substitutions to Gly and Tyr were also observed more times in the COSMIC database than His and are thus strong dominant-negative candidates.

Not all putative PTEN dominant-negative variants occur at active site residues. Pro38Ser, which is adjacent to the active site, likely exerts its effect by perturbing the active site conformation enough to render it catalytically dead, without destabilizing the global folding of the protein. Beyond perturbing the active site, other mechanisms, such as aberrant subcellular localization, could confer dominant-negative effects. One candidate can be found in the T98G glioblastoma cell line which encodes a PTEN Leu42Arg variant. Leu42Arg has a normal abundance and intact phosphatase activity but aberrant membrane localization [49]. While Leu42Arg exhibited an intermediate activity score, it was absent in our abundance data, so we were unable to incorporate this variant into our analysis. However, T98G cells exhibit increased pAkt levels (Fig. 4D), suggesting that Leu42Arg may possess a dominant-negative function that does not directly impact the active site. Notably, Cas9-based reversion of only one of the two Leu42Arg alleles in T98G cells did not alter steady-state Akt T308 phosphorylation, supporting its purported dominant-negative activity [50].

PTEN homodimerization may explain why some abundant, lipid phosphatase defective variants can act as dominant negatives while others cannot. In particular, lipid phosphatase defective variants that fail to homodimerize would not be able to bind to WT PTEN and thus could not exert dominant-negative activity. The majority of previously known PTEN-dominant negative variants substitute conserved residues in the catalytic motif, including Cys124, Asp92, and Arg130. These variants effectively eliminate enzymatic function while potentially having little effect on protein stability and three-dimensional conformation. Thus, they would be able to dimerize with WT PTEN protein and dominantly suppress their function [40]. Importantly, the large-scale variant effect datasets alone were not sufficient to identify dominant-negative PTEN variants. Not all abundant but inactive variants exhibited dominant-negative activity, as five of the variants we tested did not elicit increased pAkt levels. Thus, comparing the variant effect data to cancer genomics data is likely the most effective approach for highlighting potential dominant-negative variants. While it is impossible to accurately estimate what fraction of abundant but inactive PTEN variants will be dominant negatives, we believe it will likely be no more than the ~13% in the panel we tested and possibly much less.

The combined analysis also illustrates the differing strengths of each assay, even when considered in isolation. The yeast PTEN activity assay directly assesses catalytic activity, thus identifying a large swath of abundant but inactive variants that would be missed with the abundance assay. The abundance assay can capture subtle decreases to protein abundance associated with hypomorphic variants, potentially outside of the dynamic range of the yeast activity assay (Fig. 2C). This is consistent with what is currently known about functionally distinct PTEN variant groups in the literature, wherein gross reductions in PTEN activity are more often associated with cancer and severe cases of PHTS, while partial losses in protein stability and subtler reductions to function are associated with milder presentations observed with autism spectrum disorder and developmental disabilities [33, 51–53]. Thus, each assay provides complementary experimental readouts that have together improved our ability to delineate how PTEN genotype impacts phenotype.

As useful as they are, large-scale functional data have many limitations. For example, seven variants are classified as abundant and active in our analysis but are also listed as pathogenic in ClinVar. One of these is His93Arg, which is known to remain abundant and partially active, but with altered substrate specificity due to changes in the phospholipid-binding site [54]. The His93Arg phosphatase activity defect was not observed in yeast [55], suggesting context-specific differences in assay readout. Furthermore, different assays may give results with dissimilar dynamic ranges, and care must be taken when comparing their results. For example, the fluorescence readings from the EGFP-fused variants in VAMP-seq likely have the highest dynamic range slightly below the abundance of the WT protein. In contrast, the yeast-based rescue assay depends upon PTEN enzymatic activity, and the dynamic range of the assay is likely highest at low levels of PTEN phosphatase activity, far less than the activity of WT. Accordingly, a large fraction of variants exhibited WT-like yeast rescue activity but low abundance (Fig. 2A, orange), revealing an almost right angle in populating the scatterplot, rather than a straight line along the diagonal. A comparison with a range of individually assessed PTEN abundance variants confirmed this pattern of relationship (Fig. 2B). For different proteins and assays, each analogous plot will likely differ, and thus, care must be taken when integrating multiple large-scale variant effect datasets and interpreting what they mean for protein-specific relationship between abundance and activity. In fact, a recent comparative analysis of yeast-based enzymatic activity and mammalian intracellular abundance of CYP2C9 revealed the opposite pattern, where there were many abundant variants with little detectable enzymatic activity, suggesting that the sensitivities of the two assays had flipped in this case [26].

In conclusion, as we and others have shown, integrative analysis of multiple large-scale variant effect datasets, each measuring different properties of a single protein, can yield new insights into protein function, protein structure and disease [24, 25, 56–58]. We anticipate that as multiplexed assays are more widely deployed, such integrative analyses will become commonplace. As such, improved methods for integrating variant effect datasets, as well as tools for analyzing these datasets in the context of structural, clinical, and evolutionary information, are needed.

## Conclusions

We showed that multidimensional, large-scale variant functional data, when paired with public cancer genomics datasets and follow-up assays, can reveal how protein variants impact protein function and illuminate mechanisms by which disease-associated variants act. We created an improved dataset for PTEN intracellular abundance and integrated it with an existing large-scale PTEN phosphatase activity dataset to separate 4178 PTEN variants into four distinct subsets based on their abundance and activity, and found that disease-associated germline variants tended to be loss of activity variants, with the majority also reduced in abundance. For PTEN somatic variants, we found that those with losses of activity and abundance were uniformly enriched across cancers, while loss of activity only variants were more variably enriched. Using follow-up assays, we found that two of these variants exhibited increased pAkt signaling consistent with a previously unappreciated dominant-negative activity of these variants. These findings highlight the importance of collecting and integrating multiple large-scale variant functional datasets for important disease genes like PTEN to provide better insights into how protein variants contribute to disease.

## Supplementary Information

**Additional file 1: Fig S1.** PTEN variant coverage by the original and second libraries. **Fig S2.** Filtering scheme for scoring multiple datasets. **Fig S3.** Comparison of abundance scores from the original and composite datasets. **Fig S4.** Comparison of new abundance scores with previously imputed values. **Fig S5.** Unmodified western blot exposures used to assess PTEN variant dominant negative activity. **Table S1.** Primer sequences for generating individual PTEN variants. **Table S2.** Statistics for new VAMP-seq cell sorting replicates.

**Additional file 2: Table S3.** Tab separated value data file containing the full set of variant abundance scores used in the analysis.

## Data Availability

The datasets supporting the conclusions of this article are available in various repositories: The raw Illumina sequencing files can be accessed at the NCBI Gene Expression Omnibus (GEO) repository under accession number GSE159469, https://www.ncbi.nlm.nih.gov/geo/query/acc.cgi?acc=GSE159469 [59]. All other data types along with an R markdown script recreating the analysis from the datafiles can be found at http://www.github.com/matreyeklab/pten_composite [17]. A data table of all PTEN variant abundance scores is included as Additional file 2: Table S3.

## Abbreviations

MAVEs: Multiplex assays of variant effects
DMS: Deep mutational scanning
PHTS: PTEN Hamartoma Tumor Syndrome
VAMP-seq: Variant abundance by massively parallel sequencing
SNVs: Single-nucleotide variation
VUS: Variants of uncertain significance
TCGA: The Cancer Genome Atlas
ASD: Autism spectrum disorder
FACS: Fluorescence-activated cell sorting
pAKT: Akt phosphorylated at Thr308
CCLE: Cancer Cell Line Encyclopedia

## References

[1] Karczewski KJ, Francioli LC, Tiao G, Cummings BB, Alföldi J, Wang Q (2020). The mutational constraint spectrum quantified from variation in 141,456 humans. Nature..

[2] Weinstein JN, Collisson EA, Mills GB, Shaw KRM, Ozenberger BA, Ellrott K (2013). The cancer genome atlas pan-cancer analysis project. Nat Genet..

[3] Landrum MJ, Lee JM, Riley GR, Jang W, Rubinstein WS, Church DM (2014). ClinVar: public archive of relationships among sequence variation and human phenotype. Nucleic Acids Res..

[4] Starita LM, Ahituv N, Dunham MJ, Kitzman JO, Roth FP, Seelig G (2017). Variant interpretation: functional assays to the rescue. Am J Hum Genet..

[5] Gasperini M, Starita L, Shendure J (2016). The power of multiplexed functional analysis of genetic variants. Nat Protoc..

[6] Lee Y-R, Chen M, Pandolfi PP (2018). The functions and regulation of the PTEN tumour suppressor: new modes and prospects. Nat Rev Mol Cell Biol..

[7] Milella M, Falcone I, Conciatori F, Cesta Incani U, Del Curatolo A, Inzerilli N (2015). PTEN: multiple functions in human malignant tumors. Front Oncol..

[8] Hasle N, Matreyek KA, Fowler DM (2019). The impact of genetic variants on PTEN molecular functions and cellular phenotypes. Cold Spring Harb Perspect Med..

[9] Matreyek KA, Starita LM, Stephany JJ, Martin B, Chiasson MA, Gray VE (2018). Multiplex assessment of protein variant abundance by massively parallel sequencing. Nat Genet..

[10] Mighell TL, Evans-Dutson S, O’Roak BJ (2018). A saturation mutagenesis approach to understanding PTEN lipid phosphatase activity and genotype-phenotype relationships. Am J Hum Genet..

[11] Jepsen MM, Fowler DM, Hartmann-Petersen R, Stein A, Lindorff-Larsen K. Classifying disease-associated variants using measures of protein activity and stability. bioRxiv. 2019. 10.1101/688234.

[12] Mighell TL, Thacker S, Fombonne E, Eng C, O’Roak BJ (2020). An integrated deep-mutational-scanning approach provides clinical insights on PTEN genotype-phenotype relationships. Am J Hum Genet..

[13] Gibson DG, Young L, Chuang R-Y, Venter JC, Hutchison CA, Smith HO (2009). Enzymatic assembly of DNA molecules up to several hundred kilobases. Nat Methods..

[14] Aronesty E. ea-utils: command-line tools for processing biological sequencing data. Github. https://github.com/ExpressionAnalysis/ea-utils. 2011.

[15] Rubin AF, Gelman H, Lucas N, Bajjalieh SM, Papenfuss AT, Speed TP (2017). A statistical framework for analyzing deep mutational scanning data. Genome Biol..

[16] Matreyek KA, Stephany JJ, Chiasson MA, Hasle N, Fowler DM (2020). An improved platform for functional assessment of large protein libraries in mammalian cells. Nucleic Acids Res..

[17] Matreyek KA (2021). PTEN_composite: PTEN fill-in abundance DMS and integration with yeast activity scores Github.

[18] Abrahams BS, Arking DE, Campbell DB, Mefford HC, Morrow EM, Weiss LA (2013). SFARI Gene 2.0: a community-driven knowledgebase for the autism spectrum disorders (ASDs). Mol Autism.

[19] Taliun D, Harris DN, Kessler MD, Carlson J, Szpiech ZA, Torres R (2021). Sequencing of 53,831 diverse genomes from the NHLBI TOPMed Program. Nature..

[20] Cerami E, Gao J, Dogrusoz U, Gross BE, Sumer SO, Aksoy BA (2012). The cBio cancer genomics portal: an open platform for exploring multidimensional cancer genomics data. Cancer Discov..

[21] Gao J, Aksoy BA, Dogrusoz U, Dresdner G, Gross B, Sumer SO (2013). Integrative analysis of complex cancer genomics and clinical profiles using the cBioPortal. Sci Signal..

[22] AACR Project GENIE Consortium (2017). AACR project GENIE: powering precision medicine through an international consortium. Cancer Discov..

[23] Matreyek KA, Stephany JJ, Fowler DM (2017). A platform for functional assessment of large variant libraries in mammalian cells. Nucleic Acids Res..

[24] Suiter CC, Moriyama T, Matreyek KA, Yang W, Scaletti ER, Nishii R (2020). Massively parallel variant characterization identifies NUDT15 alleles associated with thiopurine toxicity. Proc Natl Acad Sci U S A..

[25] Chiasson MA, Rollins NJ, Stephany JJ, Sitko KA, Matreyek KA, Verby M (2020). Multiplexed measurement of variant abundance and activity reveals VKOR topology, active site and human variant impact. Elife.

[26] Amorosi CJ, Chiasson MA, McDonald MG, Wong LH, Sitko KA, Boyle G (2021). Massively parallel characterization of CYP2C9 variant enzyme activity and abundance. Am J Hum Genet..

[27] Gray VE, Hause RJ, Luebeck J, Shendure J, Fowler DM (2018). Quantitative missense variant effect prediction using large-scale mutagenesis data. Cell Syst..

[28] Weile J, Sun S, Cote AG, Knapp J, Verby M, Mellor JC (2017). A framework for exhaustively mapping functional missense variants. Mol Syst Biol..

[29] Georgescu MM, Kirsch KH, Kaloudis P, Yang H, Pavletich NP, Hanafusa H (2000). Stabilization and productive positioning roles of the C2 domain of PTEN tumor suppressor. Cancer Res..

[30] Han SY, Kato H, Kato S, Suzuki T, Shibata H, Ishii S (2000). Functional evaluation of PTEN missense mutations using in vitro phosphoinositide phosphatase assay. Cancer Res..

[31] Fricano-Kugler CJ, Getz SA, Williams MR, Zurawel AA, DeSpenza T, Frazel PW (2018). Nuclear excluded autism-associated phosphatase and tensin homolog mutations dysregulate neuronal growth. Biol Psychiatry..

[32] He X, Thacker S, Romigh T, Yu Q, Frazier TW, Eng C (2015). Cytoplasm-predominant Pten associates with increased region-specific brain tyrosine hydroxylase and dopamine D2 receptors in mouse model with autistic traits. Mol Autism..

[33] Spinelli L, Black FM, Berg JN, Eickholt BJ, Leslie NR (2015). Functionally distinct groups of inherited PTEN mutations in autism and tumour syndromes. J Med Genet..

[34] Choi SW, Lee Y, Shin K, Koo H, Kim D, Sa JK (2021). Mutation-specific non-canonical pathway of PTEN as a distinct therapeutic target for glioblastoma. Cell Death Dis..

[35] Ngeow J, He X, Mester JL, Lei J, Romigh T, Orloff MS (2012). Utility of PTEN protein dosage in predicting for underlying germline PTEN mutations among patients presenting with thyroid cancer and Cowden-like phenotypes. J Clin Endocrinol Metab..

[36] Furnari FB, Lin H, Huang HS, Cavenee WK (1997). Growth suppression of glioma cells by PTEN requires a functional phosphatase catalytic domain. Proc Natl Acad Sci U S A..

[37] Zhou X-P, Marsh DJ, Morrison CD, Chaudhury AR, Maxwell M, Reifenberger G (2003). Germline inactivation of PTEN and dysregulation of the phosphoinositol-3-kinase/Akt pathway cause human Lhermitte-Duclos disease in adults. Am J Hum Genet..

[38] Wong CW, Wang Y, Liu T, Li L, Cheung SKK, Or PM-Y (2020). Autism-associated PTEN missense mutation leads to enhanced nuclear localization and neurite outgrowth in an induced pluripotent stem cell line. FEBS J..

[39] Mester JL, Ghosh R, Pesaran T, Huether R, Karam R, Hruska KS (2018). Gene-specific criteria for PTEN variant curation: recommendations from the ClinGen PTEN Expert Panel. Hum Mutat..

[40] Papa A, Wan L, Bonora M, Salmena L, Song MS, Hobbs RM (2014). Cancer-associated PTEN mutants act in a dominant-negative manner to suppress PTEN protein function. Cell..

[41] Wang H, Karikomi M, Naidu S, Rajmohan R, Caserta E, Chen H-Z (2010). Allele-specific tumor spectrum in pten knockin mice. Proc Natl Acad Sci U S A..

[42] Barretina J, Caponigro G, Stransky N, Venkatesan K, Margolin AA, Kim S (2012). The Cancer Cell Line Encyclopedia enables predictive modelling of anticancer drug sensitivity. Nature..

[43] Smith IN, Thacker S, Seyfi M, Cheng F, Eng C (2019). Conformational dynamics and allosteric regulation landscapes of germline PTEN mutations associated with autism compared to those associated with cancer. Am J Hum Genet..

[44] Aguissa-Touré A-H, Li G (2012). Genetic alterations of PTEN in human melanoma. Cell Mol Life Sci..

[45] Lee JO, Yang H, Georgescu MM, Di Cristofano A, Maehama T, Shi Y (1999). Crystal structure of the PTEN tumor suppressor: implications for its phosphoinositide phosphatase activity and membrane association. Cell..

[46] Tate JG, Bamford S, Jubb HC, Sondka Z, Beare DM, Bindal N (2019). COSMIC: the catalogue of somatic mutations in cancer. Nucleic Acids Res..

[47] Xiao Y, Chia JYC, Gajewski JE, Lio DSS, Mulhern TD, Zhu H-J (2007). PTEN catalysis of phospholipid dephosphorylation reaction follows a two-step mechanism in which the conserved aspartate-92 does not function as the general acid—mechanistic analysis of a familial Cowden disease-associated PTEN mutation. Cell Signal..

[48] Chia JY-C, Gajewski JE, Xiao Y, Zhu H-J, Cheng H-C (2010). Unique biochemical properties of the protein tyrosine phosphatase activity of PTEN—demonstration of different active site structural requirements for phosphopeptide and phospholipid phosphatase activities of PTEN. Biochimica et Biophysica Acta (BBA) - Proteins and Proteomics..

[49] Nguyen H-N, Yang J-M, Rahdar M, Keniry M, Swaney KF, Parsons R (2015). A new class of cancer-associated PTEN mutations defined by membrane translocation defects. Oncogene..

[50] Hill VK, Kim J-S, James CD, Waldman T (2017). Correction of PTEN mutations in glioblastoma cell lines via AAV-mediated gene editing. PLoS One..

[51] Portelli S, Barr L, de Sá AGC, Pires DEV, Ascher DB (2021). Distinguishing between PTEN clinical phenotypes through mutation analysis. Comput Struct Biotechnol J..

[52] Johnston SB, Raines RT (2015). Conformational stability and catalytic activity of PTEN variants linked to cancers and autism spectrum disorders. Biochemistry..

[53] Leslie NR, Longy M (2016). Inherited PTEN mutations and the prediction of phenotype. Semin Cell Dev Biol..

[54] Redfern RE, Daou M-C, Li L, Munson M, Gericke A, Ross AH (2010). A mutant form of PTEN linked to autism. Protein Sci..

[55] Rodríguez-Escudero I, Oliver MD, Andrés-Pons A, Molina M, Cid VJ, Pulido R (2011). A comprehensive functional analysis of PTEN mutations: implications in tumor- and autism-related syndromes. Hum Mol Genet..

[56] Starita LM, Young DL, Islam M, Kitzman JO, Gullingsrud J, Hause RJ (2015). Massively parallel functional analysis of BRCA1 RING domain variants. Genetics..

[57] Park J, Selvam B, Sanematsu K, Shigemura N, Shukla D, Procko E (2019). Structural architecture of a dimeric class C GPCR based on co-trafficking of sweet taste receptor subunits. J Biol Chem..

[58] Andrews B, Fields S (2020). Distinct patterns of mutational sensitivity for λ resistance and maltodextrin transport in Escherichia coli LamB. Microb Genom..

[59] Matreyek KA, Fowler DM (2021). Multidimensional PTEN missense variant analysis reveals variant subgroups including potential dominant negatives.

