# Supplementary material for: Integrating thousands of PTEN variant activity and abundance measurements reveals variant subgroups and new dominant negatives in cancers
Source: Genome Med. 2021 Oct 14;13:165. doi: 10.1186/s13073-021-00984-x (PMC8518224; doi:10.1186/s13073-021-00984-x)
Supplement: Supplementary file 1 — Additional file 1: Fig S1. PTEN variant coverage by the original and second libraries. Fig S2. Filtering scheme for scoring multiple datasets. Fig S3. Comparison of abundance scores from the original and composite datasets. Fig S4. Comparison of new abundance scores with previously imputed values. Fig S5. Unmodified western blot exposures used to assess PTEN variant dominant negative activity. Table S1. Primer sequences for generating individual PTEN variants. Table S2. Statistics for new VAMP-seq cell sorting replicates. [file 13073_2021_984_MOESM1_ESM.docx]

**
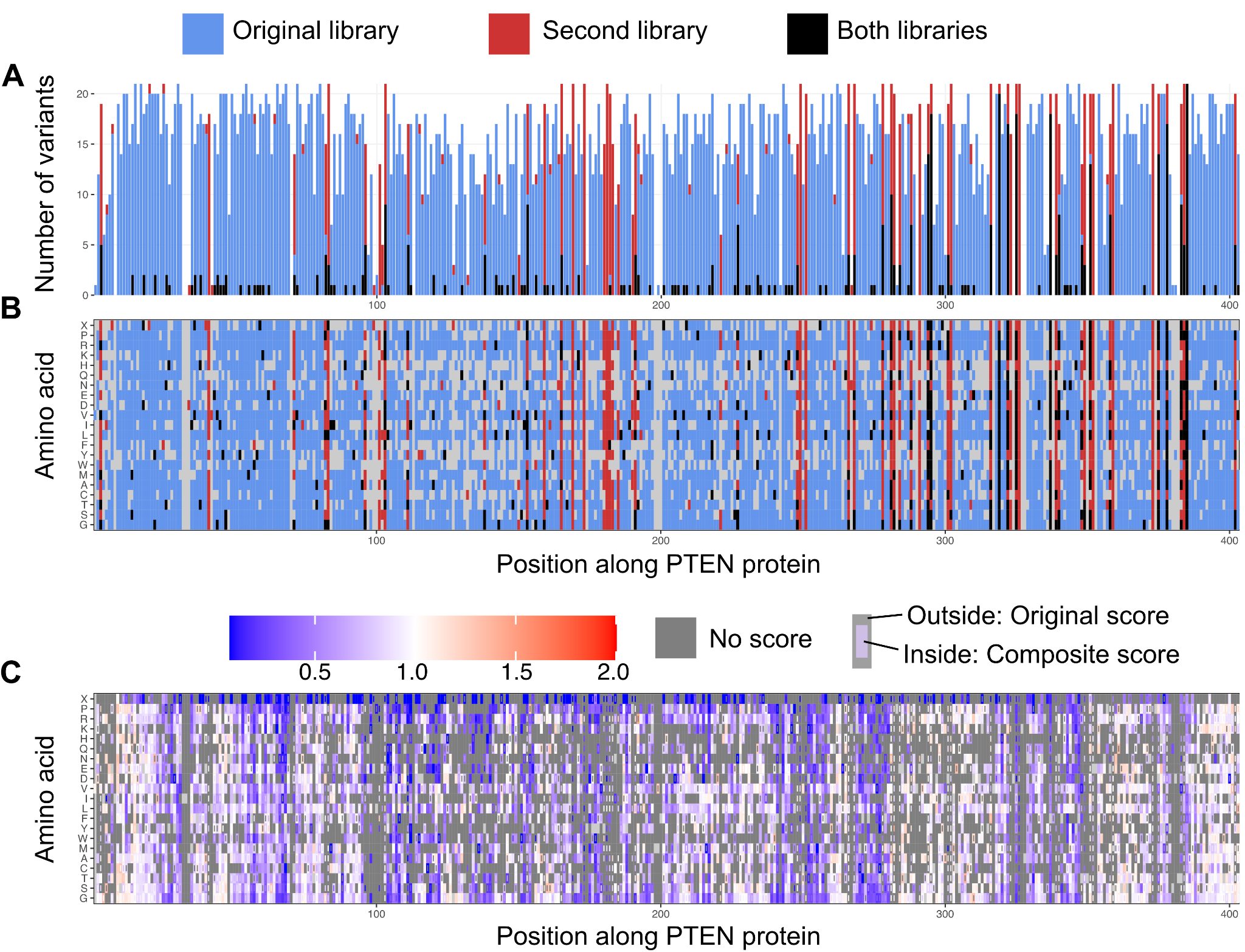
**

**Fig S1.** **PTEN variant coverage by the original and second libraries.** **A)** A bar graph showing how many variants were observed at each position of PTEN for each library, including synonymous and nonsense variants. Blue bars are variants found in the original library, red bars are variants found in the second library, and black bars denote variants found in both. **B)** A sequence-function map showing the variants that were present in one or both libraries. Tiles are colored according to the scheme described for panel A, except for the grey tiles which denote variants that were not observed in either library. **C)** Sequence-function heatmap showing the abundance scores for PTEN variants (y-axis) across the length of the PTEN protein (x-axis). The color on the perimeter of each tile shows the abundance score from the original study, while the color of the inset of each tile shows the score from the composite dataset.


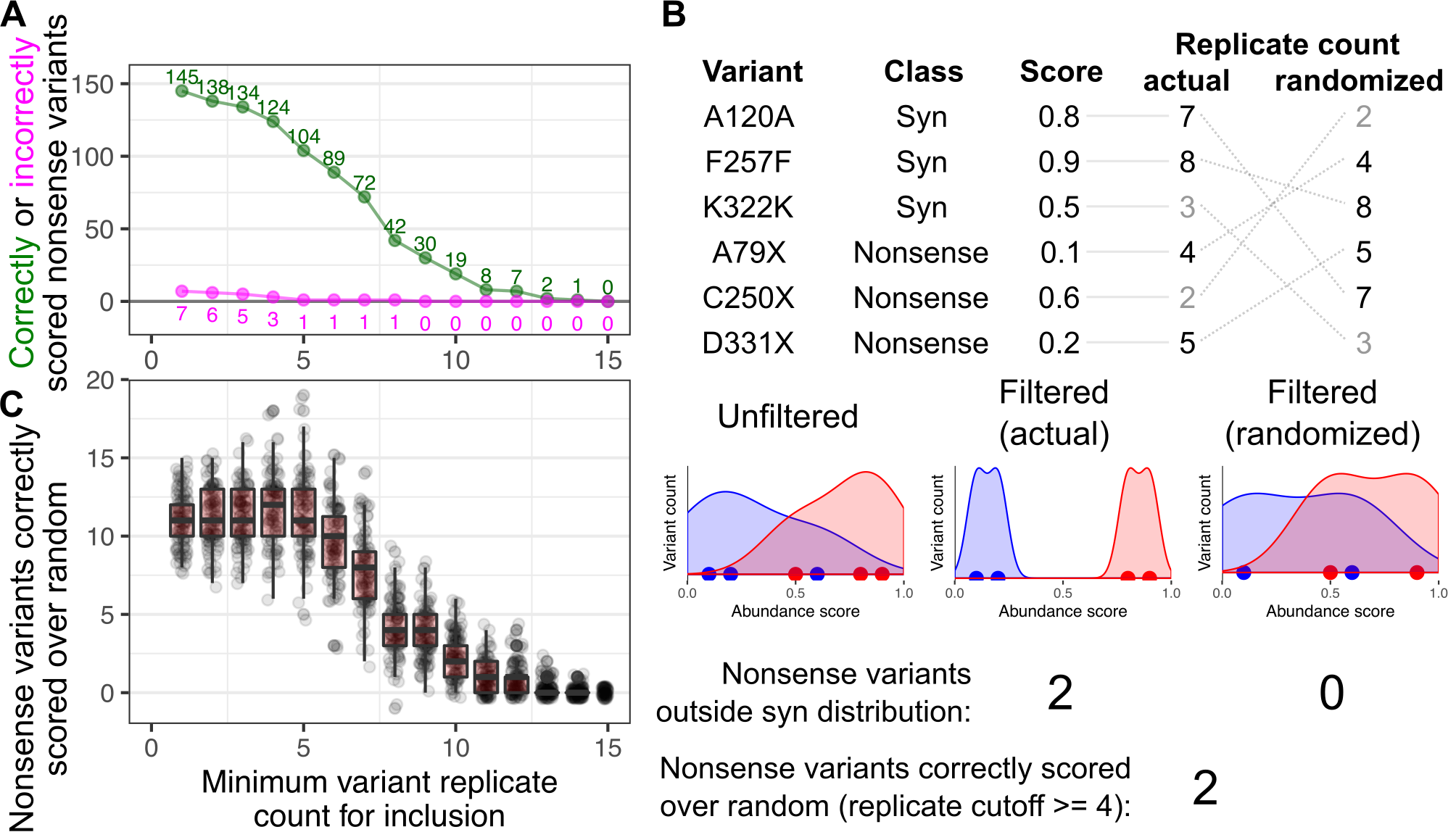


**Fig S2. Filtering scheme for scoring multiple datasets.** Knowing that scores from infrequently measured variants would likely be inaccurate, we sought a filtering scheme that would adapt to future datasets and maximize accuracy while minimizing the number of informative data points removed by the filter. We tested a replicate filter wherein a variant had to be observed in a minimal number of replicates to pass the filter, using nonsense variants at positions 50 through 350 (variants of known low abundance), and determined how many were correctly scored at each possible value of the replicate filter. To perform this test, for each replicate filter value, we determined the threshold value of the lowest 5% of synonymous variant scores that passed the replicate filter, and for each nonsense variant that also passed the replicate filter, determined whether the upper bound of the 95% confidence interval of that nonsense variant score crossed this threshold. If not, then it was deemed correctly scored. Nonsense variants that overlapped with the synonymous distribution were deemed incorrectly scored. **A)** Increasing the replicate filter reduced the number of incorrectly scored nonsense variants, but also concomitantly reduced the number of correctly scored nonsense variants. **B)** To find the maximally informative replicate filter value that gave the greatest signal over noise, we created a resampling scheme, where we performed the same test, but first randomized the score associations with the number of replicates in which the score was a result of. We then subtracted the number of correctly scored nonsense variants upon randomization from the number of correctly scored nonsense variants when replicate counts were kept linked to the scores. **C)** Results of replicate filtering test. Each point denotes the number of correctly scored nonsense variants over a randomized test set, with box plots denoting summary statistics. Randomization tests were run 100 times for each replicate filter cutoff value. This process showed that, for our dataset, a replicate filter value of 4 gave us the best chance at correctly scoring the gold-standard nonsense variants, and presumably, the missense variants in the dataset as well.


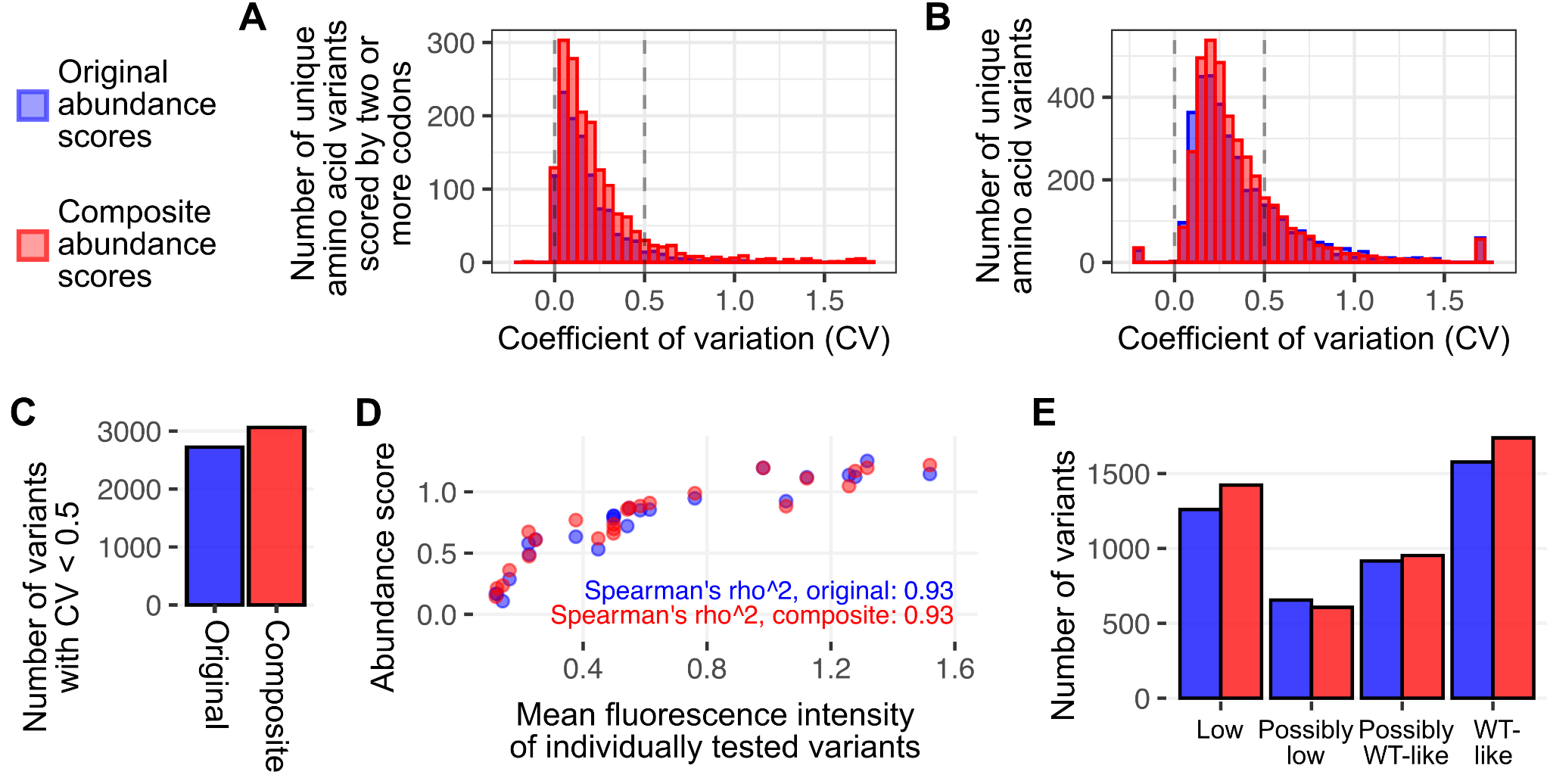


**Fig S3.** **Comparison of abundance scores from the original and composite datasets. A)** Coefficients of variation for scores calculated as a mean of the scores from two or more codons encoding a given amino acid variant, with the values calculated for the original (blue) or composite (red) datasets. **B)** Coefficients of variation of abundance scores calculated from amino acid variants observed in four or more replicates from the original or composite datasets. **C)** Summary of the total number of amino acid variants in each dataset with a coefficient of variation less than 0.5 **D)** Comparison of the correlation between the original or composite abundance scores, and the geometric mean of the mean fluorescence intensities of amino acid variants tested individually in the original VAMP-seq study. **E)** The number of amino acid variants in each abundance class, in the original or composite datasets.


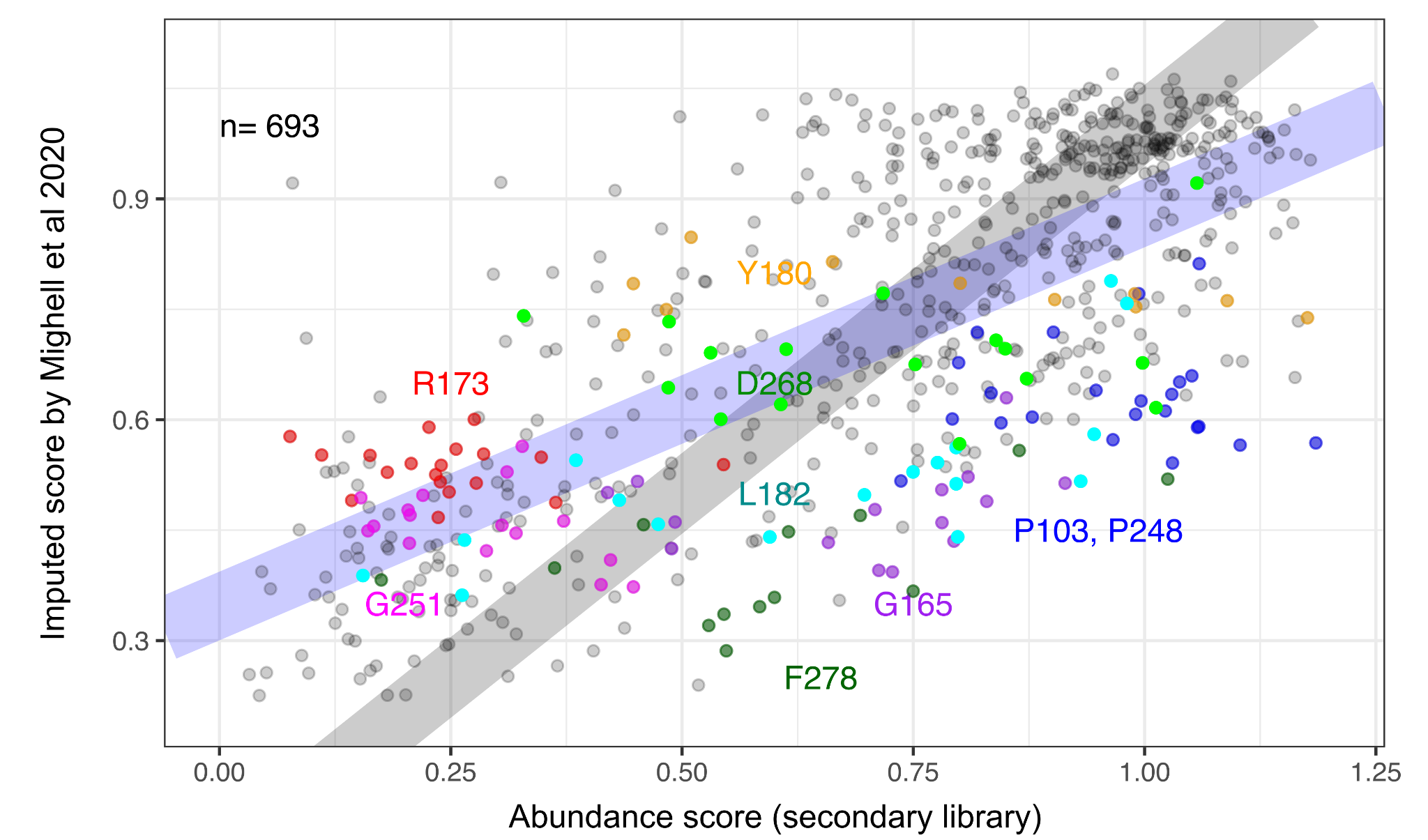


**Fig S4. Comparison of new abundance scores with previously imputed values.** Scatter plot of calculated abundance scores and imputed abundance scores from Mighell *et al*, 2020. Grey line: ideal correlation with a slope of 1 and intercept of 0. Purple line: Observed correlation with slope of 0.53 and intercept of 0.35. Positions that were inaccurately imputed by the Mighell *et al* study are highlighted and labeled with different colors.


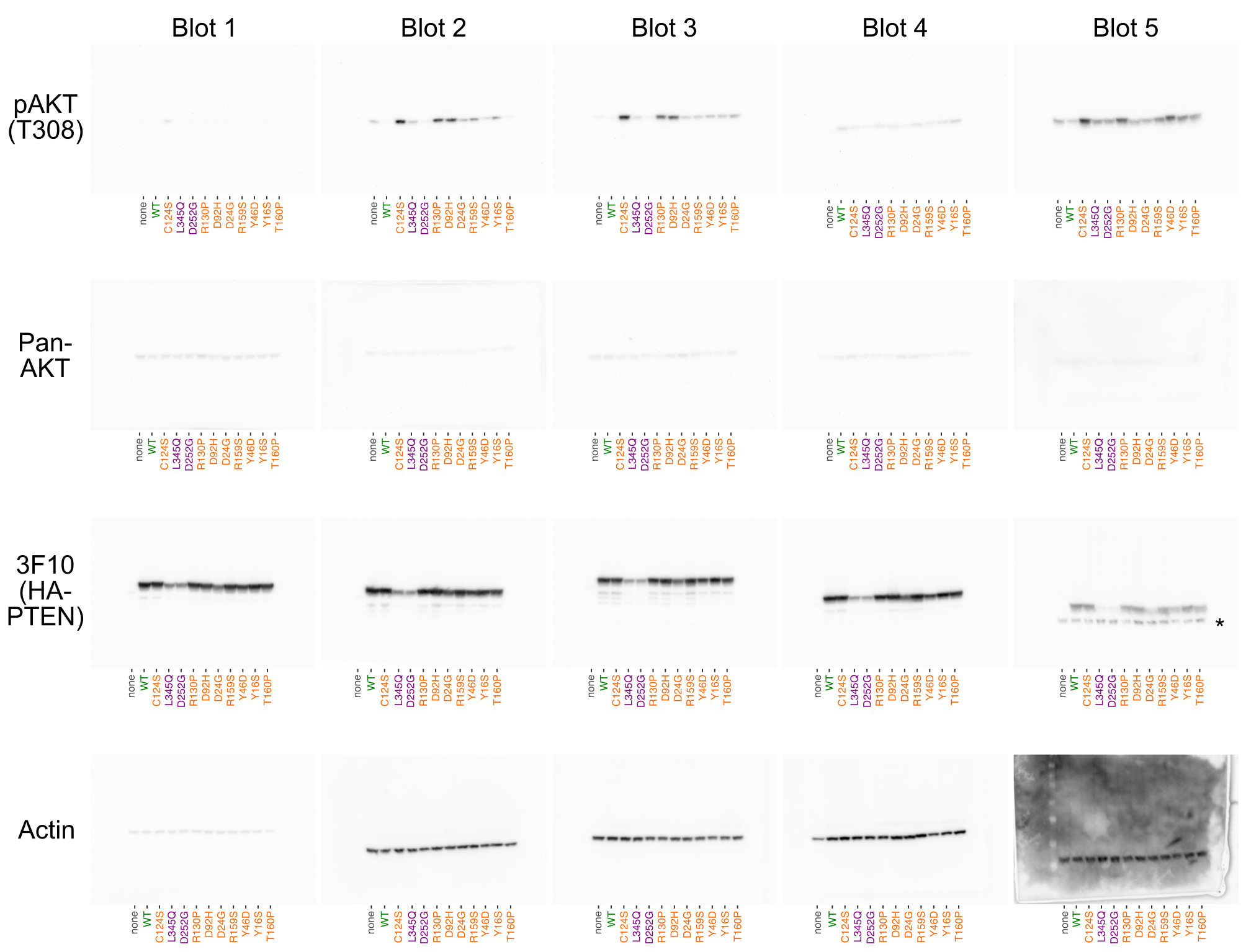


**Fig S5. Unmodified western blot exposures used to assess PTEN variant dominant negative activity.** Cells overexpressing WT or various PTEN variants were lysed on five different occasions, fractionated on a SDS-PAGE gel, and protein compositions were assessed using Western blotting. The membranes were sequentially challenged with antibody detected pAKT (T308), Pan-AKT, the HA-tag associated with overexpressed PTEN, and Beta-actin. All images are unscaled (utilizing grayscale values ranging from 0 to 65535) with a gamma factor of 1. The asterisk denotes residual signal for the actin protein which remained visible in the HA-tag Western for blot 5. Lanes are ordered as in Figure 4.

**Table S1. Primer sequences for generating individual PTEN variants**

| Name | Sequence | Purpose |
| --- | --- | --- |
| KAM2610 | aaagggacCaactggtgtaatgatatgtgcata | Primer for making the R130P PTEN variant |
| KAM2611 | caccagttGgtccctttccagctttacag | Primer for making the R130P PTEN variant |
| KAM2612 | cttttgaaCaccataacccaccacagc | Primer for making the D92H PTEN variant |
| KAM2613 | gttatggtGttcaaaaggatattgtgcaactct | Primer for making the D92H PTEN variant |
| KAM2614 | cgacttagGcttgacctatatttatccaaacattattgc | Primer for making the D24G PTEN variant |
| KAM2615 | aggtcaagCctaagtcgaatccatcctcttg | Primer for making the D24G PTEN variant |
| KAM2616 | gaagtaagCaccagagacaaaaagggagt | Primer for making the R159S PTEN variant |
| KAM2617 | tctctggtGcttacttccccatagaaatctagg | Primer for making the R159S PTEN variant |
| KAM2618 | aaggcgtaGacaggaacaatattgatgatgtagtaa | Primer for making the Y46D PTEN variant |
| KAM2619 | gttcctgtCtacgccttcaagtctttctgc | Primer for making the Y46D PTEN variant |
| KAM2620 | aaggagatCtcaagaggatggattcgactt | Primer for making the Y16S PTEN variant |
| KAM2621 | cctcttgaGatctccttttgtttctgctaacg | Primer for making the Y16S PTEN variant |
| KAM2622 | aagtaaggCccagagacaaaaagggagtaac | Primer for making the T160P PTEN variant |
| KAM2623 | gtctctggGccttacttccccatagaaatctag | Primer for making the T160P PTEN variant |

**Table S2. Statistics for new VAMP-seq cell sorting replicates**

| Replicate | cells_bin1 | cells_bin2 | cells_bin3 | cells_bin4 | cells_total |
| --- | --- | --- | --- | --- | --- |
| 1 | 408615 | 427051 | 429395 | 422204 | 1687265 |
| 2 | 440202 | 454634 | 436812 | 462689 | 1794337 |
| 3 | 469385 | 467407 | 465317 | 441061 | 1843170 |
| 4 | 454165 | 494878 | 470787 | 467532 | 1887362 |
| 5 | 660883 | 613577 | 654624 | 633326 | 2562410 |
| 6 | 568809 | 603835 | 585759 | 534346 | 2292749 |
| 7 | 619264 | 605899 | 614521 | 575372 | 2415056 |
